# Enhanced efficiency of MS/MS all-ion fragmentation for non-targeted analysis of trace contaminants in surface water using multivariate curve resolution and data fusion

**DOI:** 10.1007/s00216-023-05102-x

**Published:** 2024-01-11

**Authors:** Maryam Vosough, Amir Salemi, Sarah Rockel, Torsten C. Schmidt

**Affiliations:** 1 Instrumental Analytical Chemistry and Centre for Water and Environmental Research (ZWU), University of Duisburg-Essen, Universitätsstr. 5, Essen, 45141 Germany; 2 Department of Clean Technologies, Chemistry and Chemical Engineering Research Center of Iran, P.O. Box 14335-186, Tehran, Iran; 3 IWW Water Centre, Moritzstr. 26, Mülheim an der Ruhr, 45476 Germany

**Keywords:** All-ion fragmentation, Data fusion, Liquid chromatography–high-resolution mass spectrometry, Surface water, Contaminants, Multivariate curve resolution, Non-targeted analysis

## Abstract

Data-independent acquisition–all-ion fragmentation (DIA-AIF) mode of mass spectrometry can facilitate wide-scope non-target analysis of contaminants in surface water due to comprehensive spectral identification. However, because of the complexity of the resulting MS^2^ AIF spectra, identifying unknown pollutants remains a significant challenge, with a significant bottleneck in translating non-targeted chemical signatures into environmental impacts. The present study proposes to process fused MS^1^ and MS^2^ data sets obtained from LC-HRMS/MS measurements in non-targeted AIF workflows on surface water samples using multivariate curve resolution-alternating least squares (MCR-ALS). This enables straightforward assignment between precursor ions obtained from resolved MS^1^ spectra and their corresponding MS^2^ spectra. The method was evaluated for two sets of tap water and surface water contaminated with 14 target chemicals as a proof of concept. The data set of surface water samples consisting of 3506 MS^1^ and 2170 MS^2^ AIF mass spectral features was reduced to 81 components via a fused MS^1^-MS^2^ MCR model that describes at least 98.8% of the data. Each component summarizes the distinct chromatographic elution of components together with their corresponding MS^1^ and MS^2^ spectra. MS^2^ spectral similarity of more than 82% was obtained for most target chemicals. This highlights the potential of this method for unraveling the composition of MS/MS complex data in a water environment. Ultimately, the developed approach was applied to the retrospective non-target analysis of an independent set of surface water samples.

**Graphical abstract:**

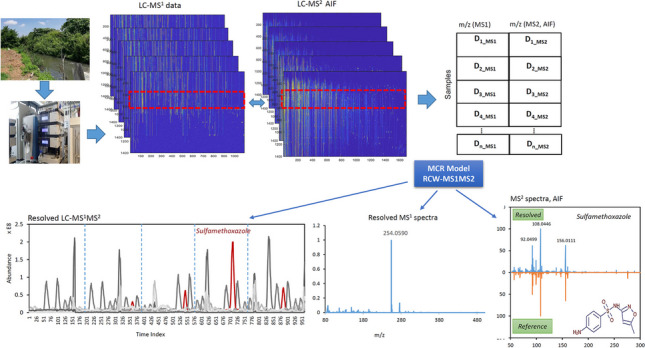

**Supplementary information:**

The online version contains supplementary material available at 10.1007/s00216-023-05102-x.

## Introduction

In recent years, the use of liquid chromatography coupled with high-resolution mass spectrometry (LC-HRMS) to analyze organic micropollutants, their transformation products, and human metabolites in environmental water samples has increased substantially. In the framework of non-target analysis (NTA), modern hybrid mass spectrometry instruments allow sensitive and comprehensive detection of hundreds of chemical compounds in environmental samples [1–3]. As an added benefit, full-scan and tandem mass spectrometry (MS/MS) data can be stored without the need for rerunning samples, enabling retrospective analysis to uncover contaminants that have never been detected before. However, while LC-HRMS/MS has provided new opportunities for NTA of organic pollutants, there are several challenges, and knowledge gaps have emerged in the environmental big data era from measurement and data processing points of view. At the end of a non-targeted workflow, for instance, identifying the preferred pollutants is an important and challenging task. When searching large compound databases for possible structures, numerous hits are usually generated, which must then be sorted by MS/MS data, retention time plausibility, and metadata provided. In fact, developing identification protocols for prioritized pollutants becomes crucial at this stage, especially with limited reference substances available. In an HRMS instrument, MS/MS can improve selectivity in annotation efforts, as MS/MS offers more annotation selectivity than does accurate mass alone. However, the search for unknowns in MS/MS or in-source fragment ion libraries is limited to the recorded spectra of reference standards, which is not sufficient for a real unknown screening and suffers from limited comparability among instruments [4]. Therefore an in silico strategy for determining unknown chemical structures by matching measured with quantitative structure–activity relationship (QSAR)-based predicted patterns from chemical databases is an alternative approach [5]. A successful MS/MS acquisition strategy could result in high-quality spectra for as many of the ions in the sample as feasible. The two most common MS^2^ data acquisition approaches are defined as data-dependent acquisition (DDA) and data-independent acquisition (DIA), for liquid chromatography combined with HRMS. In DDA, differentiation can be made between a targeted approach (defined by the user, also known as list-dependent) and MS logic (e.g., top N of maximum peak height). DDA is generally used to increase annotation confidence in a non-targeted study. However, the limited MS/MS coverage of detected MS features in DDA has spurred the development of several alternative approaches, referred to as modified DDA approach [6]. In DIA, precursor windows are sequentially isolated and fragmented within the ion trap, thereby covering all precursor ions of interest. As a result of its wider isolation window, DIA offers the advantage of not requiring any prior knowledge of precursors. However, it displays more complex MS/MS spectra. Different DIA approaches include sequential precursor ion fragmentation (MS/MS^ALL^), sequential window acquisition of all theoretical mass spectra (SWATH), and an innovative mode of acquisition known as scanning quadrupole DIA (SONAR) [7]. The MS/MS^ALL^ can be categorized into two methods, MS^E^ and all-ion fragmentation (AIF). MS^E^ is a mode that alternates between low-energy and elevated-energy scans, providing more comprehensive information about the sample. In AIF, all ions in the collision cell are fragmented without precursor ion selection. Therefore, AIF full scans combined with MS^1^ scans provide an opportunity to retroactively analyze additional compounds of interest based on hypotheses that arise in the future [8]. However, in AIF, the selectivity is lost because no precursor ion selection is used, so the links between precursors and their fragment ions become untraceable due to the complexity of the resulting MS^2^ spectra. Several deconvolution algorithms have been developed, including MS-DIAL [9], DIA-Umpire [10], R-MetaboList [11], and CorrDec (correlation-based deconvolution) [12] to link AIF parent ions to their associated fragment ions and extract the relevant pseudo-MS/MS spectra. One recent study developed an automated multi-sample-based correlation AIF workflow (MetaboAnnotatoR) for the annotation of -omics LC−MS AIF data sets [13]. In fact, research in this area is ongoing, specifically in the field of metabolomics, as well as evaluating different MS modes and data types [14]. Though these software programs offer a good starting point for NTA of water samples, they were originally developed for -omics research, and to the best of our knowledge, they have not yet been thoroughly tested for their effectiveness and functionality in NTA of water samples using the DIA approach. In fact, high-coverage non-target environmental screening presents several complications to researchers mainly due to the large diversity of environmental matrices and chemical space, the occurrence of low-intensity but highly environmentally effective contaminants, and substantial matrix effects in highly contaminated water samples [15–17]. In most of the mentioned tools, the precursor–fragment ion connection is performed by peak matching or peak intensity correlation matching across a fixed retention time region. Relying on peak shape or correlation shape-based feature tracing is highly problematic in the case of co-elution of compounds or embedded peaks. Moreover, when co-eluting compounds produce similar mass fragments, their intensity correlation is so small that it cannot be detected in deconvoluted MS^2^ spectra. Additionally, some adducts, in-source fragments, and isotopologues are not always taken into account.

In recent years, extended multivariate curve resolution–alternating least squares (MCR-ALS) combined with a binning procedure has been developed for non-target analysis [18, 19]. Data compression and matrix construction can also be conducted according to searches of regions of interest (ROI), which are regions of data points with a high density ranked by a certain "data void" [20, 21]. Following the coupling of ROI with the MCR-ALS method in metabolomics studies [22–24], the method has been utilized for non-target analysis in environmental metabolomics [25], micropollutant screening in aquatic environments [15, 26], wastewater proteomics [27], polymer degradation in aquatic environments [28], and recently in the processing of different MS acquisition modes in an non-targeted metabolomics study [29]***.*** The main strength of employing MCR-ALS in NTA studies is that, unlike most data processing strategies which are based on analyzing each *m/z* channel (feature) at a time for each sample and require alignment and finally a componentization step, MCR-ALS is based on a bilinear factor decomposition concept. Thus, all information regarding the mass features of each MCR-ALS component, such as precursor ions, their associated isotopic peaks, and adduct peaks, can be recovered at once and considered for identification purposes. Additionally, this method does not require background signal correction, since it recovers all chemicals, solvent peaks, and background signals responsible for systematic variation in the data sets. Moreover, MCR-ALS is the most flexible multi-way model for handling retention time shifts across chromatographic runs. In fact, because of the bilinear factor decomposition basis, an alignment of the retention time shift is not essentially required before performing MCR-ALS modeling, which is advantageous [30]. The reports, however, find that shift corrections and trilinearity constraints (and other constraints whenever applicable) can improve reliability and reduce uncertainty in many situations, depending on the data structure [31, 32]. Using this concept, MS^2^ AIF data across different LC runs can be compiled as matrices, and multi-way data modeling can be applied to them, in a similar way as MS^1^ full-scan data. In fact, by employing this method, a component-based profile resolution strategy rather than a feature-based deconvolution method is developed for MS^2^ spectral recovery. One further step is fusing two blocks of data sets and their data processing. This work aims to develop the concept of MCR-ALS for simultaneous decomposition of LC-MS^1^ and LC-MS^2^ data sets in AIF acquisition mode in surface water samples, in a non-targeted way. In this study, we investigate how data integration in different MS acquisition modes can facilitate unknown peak identification in samples of different complexity. In addition to the curve resolution in each individual MS measurement mode, extended MCR-ALS allows researchers to analyze them simultaneously (both row-wise and column-wise) by fusing the data. The present study is the first to report the joint processing of augmented full MS/AIF modes data for NTA of organic micropollutants in water samples using a multi-way decomposition method. The advantages of this method for a simultaneous analysis of multiple chromatographic runs include the resolution of all components having a systematic variation within a raw data set in all instrumental modes and their relative abundance, and obtaining a direct connection between each precursor ion (together with its adducts, isotopes, and fragments) and its corresponding MS/MS spectral profiles. In order to achieve this, we prepared reference spectra for 14 pharmaceuticals and hormones frequently detected in surface water, evaluated the decomposition process in individual models of MS^1^, MS^2^, and fused MS^1^-MS^2^ data, and used different validation samples by spiking the target compounds in tap water and river water samples. Moreover, the match quality scores with the reference spectra were used to evaluate the quality of resolved MS^2^ spectral profiles and clarifying some MCR challenges in this area. Ultimately, as a “real-life” application in this field, the developed approach was applied to the retrospective NTA of an independent set of surface water samples, following the classification and prioritization step. Our main objective was to implement a strategy for annotating highly prioritized chemicals based on the relevant chromatographic segments.

## Material and methods

### Chemicals, samples, and data acquisition

Authentic standards of 14 targeted chemicals including primidone, caffeine, acetaminophen, sulfamethoxazole, trimethoprim, testosterone, carbamazepine, napoxen, ibuprofen, gemfibrozil, fluoxetine, ciprofloxacin, estrone, and progesterone were purchased from Sigma-Aldrich (Saint Louis, MO, USA), and detailed information of these chemicals is provided in Table S1 (see Electronic Supplementary Material, S1). The mixed standard solutions (set I samples) were prepared in methanol at 1000 μg/L and stored at −20 °C, and different solutions of standard solutions (0.1, 0.5, 1, 10, 50, and 100 μg/L) were prepared in ultrapure water. LC-MS-grade water, LC-grade methanol, and formic acid were purchased from Merck (Darmstadt, Germany). A duplicate analysis was performed on all samples. The performance of the proposed methodology was then evaluated by spiking the mixed standard targets into two types of water samples with varying levels of matrix complexity (validation samples). To this end, tap water samples and river water samples collected from the Ruhr River south of Essen (Germany) were considered. Set II samples represent a “low-level” matrix complexity and the non-spiked and all spiked tap water samples (TW1–TW7) were directly injected into the LC-Q-Orbitrap-MS system. To generate set III samples which represent "high-level" matrix complexity, the river water sample was first extracted by solid phase extraction (SPE), and then the standards mixtures were spiked into the extracted water (RW1–RW7) with a nominal enrichment factor of 100. Details of sample preparation are presented in S2. Set II and set III samples were prepared at six-point concentrations of 14 pharmaceuticals and hormones (0.1 to 100 μg/L). Furthermore, the proposed approach was applied to an independent set of surface water samples (set IV) as part of a non-target study. Samples were collected in May 2019 at five different points in rivers of northern Iran. Details of sampling points and the laboratory and data analysis practices for set IV samples are presented in S3. All measurements were conducted using a Dionex UltiMate 3000 LC system (Thermo Scientific) hyphenated to a high-resolution accurate-mass Orbitrap mass spectrometer (Q Exactive, Thermo Scientific) with acquisition parameters and conditions described in S2 (data sets I to III) and S3 (data set IV).

### Initial data preparation and matrix arrangement

**Fig. 1:**
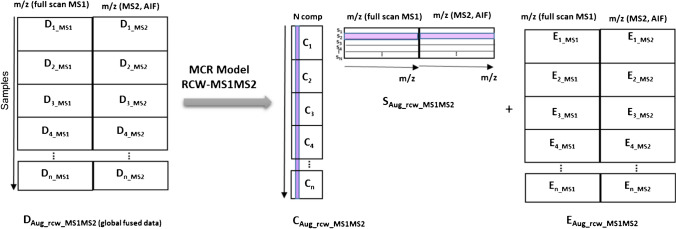
Representation of row/column-wise augmentation (RCWA) and extended MCR modeling of all samples in both MS^1^ and MS^2^ modes (one global model)

The MS^1^ and MS^2^AIF information in structure arrays was converted to peak lists (cell array of matrices containing *m/z* values and ion intensity values) using the “mzxml2peaks.m” function of the MATLAB Bioinformatics Toolbox (4.3.1.version) by setting *LevelsValue* to “1” and “2,” respectively. Then, the strategy based on the ROI approach was employed in all measured LC-HRMS/MS signals for data compression and preparing final data matrices [21–23]. Then, ROI analysis was performed on each data file's individual peak lists. In fact, by using ROIs, these initial arrays with their irregularly distributed measured *m/z* and MS instrument signal intensities can be converted into data matrices appropriate for multivariate data analysis. The ROI selection is governed by the mass intensity threshold (SNR_thr_), the *m/z* error or the mass accuracy of the spectrometer and the minimum number of occurrences of *m/z* values in consecutive scans for an “ROIpeaks” function [24], which were set at 0.01% of the maximum MS signal intensity, 0.003 amu for the Orbitrap MS analyzer and 15, respectively. At the end of this step, the matrixized data for each sample in both MS^1^ and MS^2^ modes of measurement were created. Then, according to Figs. S1, S2 and Fig. 1, different data arrangements were made to model the data sets by the extended MCR method: (a) column-wise augmentation (CWA) of all samples in each of MS^1^ or MS^2^ modes (using the “MSroiaug” function), (b) row-wise augmentation (RWA) of LC-MS^1^ and LC-MS^2^ for each sample, and (c) row-column-wise augmentation (RCWA) of all samples for both MS^1^ and MS^2^ modes. Each individual data matrix can be segmented before this step in order to simplify the further curve resolution process and localize component information within narrow time frames. If there are high background signals or if peak windows cannot be selected in which each peak is completely contained in at least one window, windows can be selected in which regions overlap.

### MCR-ALS processing of LC-MS^1^, LC-MS^2^, and fused LC-MS^1^-MS^2^ data

MCR-ALS is a well-known method that decomposes bilinear data sets into pure component profiles describing the measured variance of the system [33]. The original form of bilinear factor decomposition through MCR-ALS can be extended to a more powerful model for the decomposition of augmented data matrices and simultaneous modeling of several samples (see Fig. S1). Moreover, data fusion can be accomplished by using different spectrometric measurements to investigate a system as row-wise augmented matrices. In LC-HRMS measurements, the concept of data fusion can be adopted for a row-wise appending of data matrices in different acquisition modes of MS^1^ and MS^2^ AIF, as shown in Fig. S2 for one sample. For multiple sample analysis, row-wise fused data sets can be augmented on top of each other to provide a global data matrix as shown in Fig. 1. As a result of the inclusion of one or more of the included data matrices, these new augmented data matrices always exhibit favorable features that affect the resolution of the most complex data matrices [30].

To obtain an appropriate and meaningful data structure, data matrices in column- and row-wise data augmentations should share column vector space (MS^1^ and MS^2^ spectra) and row vector space (LC elution windows) with the other appended matrices, respectively. Therefore, while column-wise chromatographic alignment is not necessarily a prerequisite before MCR-ALS processing of RCWA data sets, row-wise augmented matrices must match before modeling begins. A misalignment condition can be checked intuitively or by individual modeling of LC-MS^1^ and LC-MS^2^ data blocks. Therefore, the retention times of resolved chromatograms of the LC-MS^1^ can be considered as the reference index for adjusting of LC-MS^2^ data. Bilinear decomposition of each global fused LC-MS^1^-MS^2^ matrix, containing *K (no. of samples)×2* matrices can also be shown as:

1 $${\textbf{D}}_{\textbf{global}}=\left[\begin{array}{c}\textbf{D}{\textbf{1}}_{\textbf{LCMS1}-\textbf{MS}\textbf{2}}\\ {}\textbf{D}{\textbf{2}}_{\textbf{LCMS1}-\textbf{MS}\textbf{2}}\\ {}\textbf{D}{\textbf{3}}_{\textbf{LCMS1}-\textbf{MS}\textbf{2}}\\ {}.\\ {}.\\ {}.\\ {}{\textbf{D}\textbf{k}}_{\textbf{LCMS1}-\textbf{MS}\textbf{2}}\end{array}\right]=\left[\begin{array}{c}\textbf{C}\textbf{1}\\ {}\textbf{C}\textbf{2}\\ {}\textbf{C}\textbf{3}\\ {}.\\ {}.\\ {}.\\ {}\textbf{C}\textbf{k}\end{array}\right]={\textbf{S}}_{\textbf{MS1}-\textbf{MS}\textbf{2}}^{\textbf{T}}+\left[\begin{array}{c}\textbf{E}{\textbf{1}}_{\textbf{LCMS1}-\textbf{MS}\textbf{2}}\\ {}\textbf{E}{\textbf{2}}_{\textbf{LCMS1}-\textbf{MS}\textbf{2}}\\ {}\textbf{E}{\textbf{3}}_{\textbf{LCMS1}-\textbf{MS}\textbf{2}}\\ {}.\\ {}.\\ {}.\\ {}{\textbf{Ek}}_{\textbf{LCMS1}-\textbf{MS}\textbf{2}}\end{array}\right]={\textbf{C}}_{\textbf{aug}}\ {\textbf{S}}_{\textbf{MS1}-\textbf{MS}\textbf{2}}^{\textbf{T}}+{\boldsymbol{E}}_{\boldsymbol{MS}\textbf{1}-\boldsymbol{MS}\textbf{2}}$$

where the rows in matrix **D**_global_ contain RWA matrices recorded in full-scan MS^1^ and MS^2^ AIF modes. **C**_aug_ contains the elution time profiles of N compounds eluted in both modes of measurements which are present in all individual sub-matrices, and ${\textbf{S}}_{\textbf{MS1}-\textbf{MS}\textbf{2}}^{\textbf{T}}$ represents the pure MS^1^ and MS^2^ spectra associated with LC profiles. **E**_**MS1−MS2**_ is the global matrix of residuals not fitted by the model. Thus, a unified MCR model can be used to obtain information regarding all involved LC profiles, taking into account different peak shapes, retention times and peak areas, as well as MS^1^ and MS^2^ profiles.

In order to obtain the best solution, it is crucial to carefully examine the number of components, initial estimate profiles, and constraints. In selecting the number of components in each **D**_**global**_, a visual inspection, incremental approach, statistical approach, or a combination of these approaches can be used. The incremental approach can be used to build MCR-ALS models with a low number of chemical components (based on singular value decomposition [SVD] or principal component analysis [PCA]) and incrementally increase the number of chemical components as the model progresses [33]. Depending on criteria such as explained variance or model stability, and reliability of resolved chromatograms, MS^1^ and MS^2^ spectra, the optimal number of components can be determined [15, 34]. The initial estimates of spectra or LC profiles were produced by the SIMPLe-to-use Interactive Self-modeling Mixture Analysis (SIMPLISMA) method [35], according to the most pure elution regions or pure *m/z* values of the involved components, respectively. Then, bilinear factor decomposition of **D**_global_ and estimation of **C**_aug_ and **S**^T^ matrices were performed by iterative least-squares minimization of the Frobenius norm of **E** (residuals/errors), under constraints of non-negativity in **C**_aug_ and **S**^T^ factor matrices, and normalization of the mass spectra of the resolved components to the maximum signal intensity equal to one [33]. Here, the data sets were modeled without background correction and in most cases without chromatographic peak alignment. However, for a few cases, after shift corrections using *icoshift* [36] and the fulfillment of the trilinear structure, the trilinearity constraint was also added to improve the quality of results. Other constraints can be implemented during the ALS optimization such as closure, unimodal, selectivity, and local rank [37], which were not considered in this work. Regarding the correspondence criterion, the default setting assumption was used in MCR-ALS analysis for adhering to a non-targeted concept. In fact, due to the clear advantage of MCR-ALS for flexible implementation of constraints, a wide variety of data sets can be processed effectively by selecting the appropriate restricting conditions, thus reducing the uncertainty associated with bilinear factor decomposition [31]. Finally, the iterative optimization is continued, until the convergence criterion is fulfilled. This criterion is based on determining the relative standard deviation of residual changes between two consecutive iterations below a predetermined threshold, (i.e., 0.1%). Furthermore, the quality of the decomposition process can be determined by the lack of fit (Lof%) and the amount of variance explained, *R*^2^, as defined by Eqs. 2 and 3:

2 $$\textrm{Lof}\ \left(\%\right)=100\ \frac{\sum_{\textrm{i}=1}^{\textrm{m}}\sum_{\textrm{j}=1}^{\textrm{n}}{\textrm{e}}_{\textrm{i},\textrm{j}}^2}{\sum_{\textrm{i}=1}^{\textrm{m}}\sum_{\textrm{j}=1}^{\textrm{n}}{\textrm{d}}_{\textrm{i},\textrm{j}}^2},{\textrm{e}}_{\textrm{i},\textrm{j}}={\textrm{d}}_{\textrm{i},\textrm{j}}-{\hat{\textrm{d}}}_{\textrm{i},\textrm{j}}$$

3 $${\textrm{R}}^2\left(\%\right)=100\ \frac{\sum_{\textrm{i}=1}^{\textrm{m}}\sum_{\textrm{j}=1}^{\textrm{n}}{\textrm{d}}_{\textrm{i},\textrm{j}}^2-\sum_{\textrm{i}=1}^{\textrm{m}}\sum_{\textrm{j}=1}^{\textrm{n}}{\textrm{e}}_{\textrm{i},\textrm{j}}^2}{\sum_{\textrm{i}=1}^{\textrm{m}}\sum_{\textrm{j}=1}^{\textrm{n}}{\textrm{d}}_{\textrm{i},\textrm{j}}^2}$$

where each *d*_ij_ shows each experimental data matrix and each *e*_ij_ is the residual element of the **E** matrix.

### Data evaluation

In the current study, the mixed standard solutions (data set I) were arranged globally (*D*_Aug-rcw-MS_^1^_MS_^2^) and subjected to extended MCR-ALS modeling to generate a robust source of reference chemical information, including resolved chromatographic profiles and MS^1^ and MS^2^ spectra and their relative abundance (Fig. 1). These results were further compared with individual modeling of CWA LC-MS^1^ and LC-MS^2^ data sets. Then, the method was used for decomposition of two validation data sets II and III. To this end, MCR models were created and evaluated for one global RCWA LC-MS^1^-MS^2^ data set. Finally, the feasibility of the method was further evaluated for individual modeling of RW fused LC-MS^1^-MS^2^ data sets for extracted river water samples enriched with target compounds with contamination levels of 100 and 10 μg/L (Fig. S2). For NTA of data set IV, the peak areas obtained via MCR modeling of CWA D_LC-MS1_ were put into a matrix whose rows and columns corresponded to the water samples and the resolved MCR-ALS components. This matrix of non-target data was subjected to multivariate methods including PCA and orthogonal partial least squares–discriminant analysis (OPLS-DA). Then, the LC-MS^1^ and LC-MS^2^ data sets for some chromatographic segments were fused to be processed by global MCR-ALS models and simultaneous decomposition of MS^1^ (including precursor ions) and MS^2^ AIF spectra.

Chromatograms were recorded in profile mode using Xcalibur software (Thermo Fisher). All chromatographic data sets were then converted into mzXML files using MSConvertGUI software [38]. Next, data files were imported into MATLAB (The MathWorks, Inc., version 9.9, 2020b, Natick, MA, USA) for further data preprocessing and postprocessing as mzXMLStruct using the “mzxmlread.m” function of the MATLAB Bioinformatics Toolbox. The calculations involving MCR-ALS were performed in MATLAB software using the MCR-ALS 2.0 toolbox available at www.mcrals.info. The *icoshift* routine was downloaded from www.models.life.ku.dk/algorithms. Chemometrics data processing (set IV samples) and prioritization of relevant contaminants were performed using PLS Toolbox version 8.9 (Eigenvector Research, Inc., Wenatchee, WA, USA) in the MATLAB computational and visualization environment. Following the decomposition process for fused LC-MS^1^-MS^2^ data sets for the standard samples, and confirming their correspondence with the extracted ion chromatograms (EICs) from the original data in Xcalibur software, the resolved MS^1^ and MS^2^ spectral profiles were used for confirmation of known non-target chemicals (validation sets) and confirmation/tentative identification of unknown contaminants in surface water samples, based on multiple lines of evidence. These include (1) a positive hit in the MS/MS libraries mzCloud (mzCloud; https://www.mzcloud.org) and PubChem (https://pubchem.ncbi.nlm.nih.gov/) and the in-house MS^2^ spectral library from experimental data in DDA, for the most intense mass fragment in the resolved MS^1^ profile (assigned to theoretical exact *m/z* [M+H]^+^ or [M+Na]^+^ precursors within a 5 ppm *m/z* error). For the validation step, due to the availability of reference MS^2^ profiles (in-house library), a similarity score or MS^2^ spectrum match was calculated as a dot product between reference MS^2^ profiles and the resolved AIF MS^2^ profiles as follows:

4 $${\textrm{MS}}^2\ \textrm{similarity}\ \left(\%\right)=100\times \frac{\sum {\left({MS}_{res}{MS}_{ref}\right)}^2}{\sum {MS_{res}}^2\sum {MS_{ref}}^2}$$

where MS_res_ and MS_ref_ are the vectors of resolved AIF MS^2^ profiles and AIF (or DDA) reference mass spectrum, respectively. Further evidence includes (2) a match between chromatographic peak shapes and retention time, and (3) availability of reference materials. To classify tentatively identified features, the scheme proposed by Schymanski was used [39].

## Results and discussion

### Decomposition of LC-HRMS/MS standard data set

Initially, each individual CWA LC-MS^1^ and LC-MS^2^ data of mixed standard solutions (set I samples) were analyzed using the MCR-ALS method. MS^1^ and MS^2^ data were simultaneously processed in the next step by fusing the matrices in a row-wise way. All models performed optimally with 25 resolved components, including 11 background signals and 14 target compounds. The percentage of explained variance and lack of fit of experimental for the global models were ≥99% and ≤6.5% (for individual models the values were ≥99% and 3–6%), respectively. Figures S3 and S4 show the resolved chromatograms for the mixed standard solution in concentration of 100 μg/L (in three models) and 0.5 μg/L in fused mode, respectively. As can be seen in these figures, all targeted compounds have been resolved well chromatographically and there is a high level of coherence between the profiles across the MS^1^, MS^2^, and MS^1^-MS^2^ mass spectral data. Furthermore, the difference in total ion currents (TICs) in LC-Q-Orbitrap for MS^1^ and MS^2^ data acquisition modes, which reflect different ion sensitivities, can be compensated for by simultaneously analyzing both MS^1^ and MS^2^ signals (see Fig. S5 as an example). With this approach, all patterns and features of both MS levels are captured in a unified chromatogram, components are identified more accurately, and the level of information is generally higher than if each block were modeled separately. A pairwise comparison between the retrieved MS^1^ and MS^2^ profiles through individual models with their hybrid model counterparts showed an excellent agreement, making the global model robust and superior to individual ones capable of recovering MS^2^ profiles and directly connecting them to relevant MS^1^ spectra (precursor ions) and chromatographic profiles. Correlation analysis between the areas under the resolved LC profiles in MS^1^-MS^2^ model and each of the MS models confirmed the high quality of chromatographic resolution of the fused model (*R*^2^ values >0.993). Moreover, based on a regression analysis between peak areas and concentrations of each chemical (between 0.1 and 100 g/L), the correlation coefficients obtained from fused data modeling ranged from 0.993 to 0.998 (*p*-value of lack-of-fit test >0.05), suggesting that the methodology can also be used as a complementary approach for quantification purposes as well (Fig. S6a and b). This study, however, focused on qualitative aspects of the results when constructing an integrative model for non-targeted analysis of RCWA LC-MS^1^-MS^2^ data sets.

**Fig. 2:**
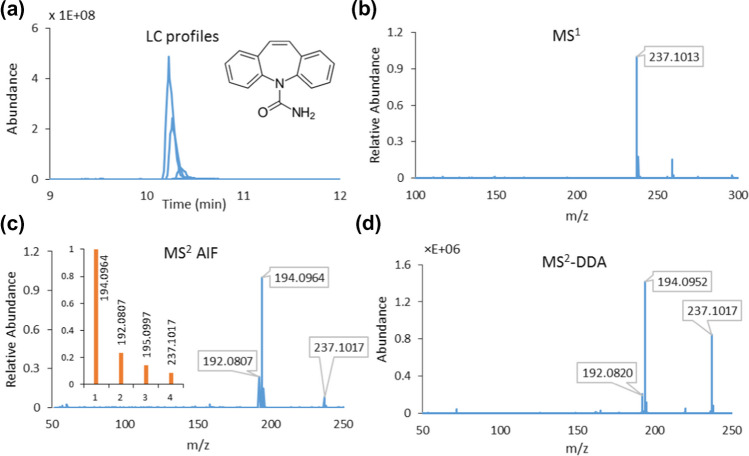
Representation of the decomposition process for carbamazepine by MCR-ALS modeling of global RCW augmented data matrices for data set I: (**a**) resolved LC profiles, (**b**) MS^1^and (**c**) MS^2^ AIF resolved spectra. The inset plot in (**c**) illustrates the relative peak heights at the characteristic EICs of pure standard carbamazepine, and (**d**) shows the MS^2^ spectrum of carbamazepine in DDA mode

**Fig. 3:**
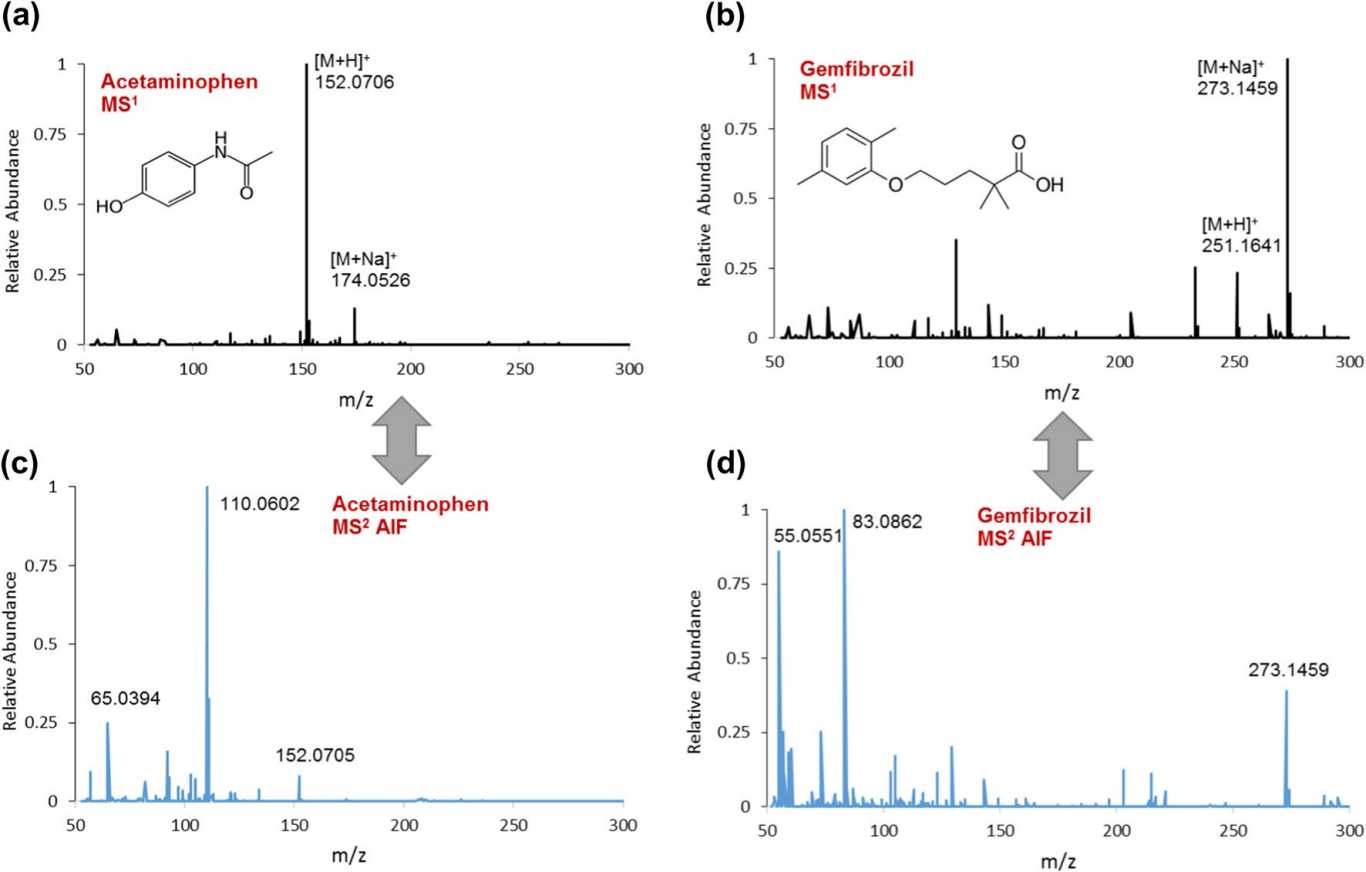
Representation of precursor ion assignment using resolved MS^1^ spectra of (**a**) acetaminophen and (**b**) gemfibrozil, and their direct links to the corresponding resolved MS^2^ AIF spectra (**c**) and (**d**) through simultaneous modeling of RCW augmented data matrices by extended MCR-ALS

Figure 2a–c shows an example of MCR-ALS modeling of global augmented data matrices for carbamazepine standard [M+H]^+^ (*m/z* 237.1013). Here, the chromatographic profiles of carbamazepine (MCR component 4) and their MS^1^ and MS^2^AIF profiles counterparts in all standard samples (with *R*^2^= 0.998) have been recovered at once. As shown in the inset of Fig. 2c, MS^2^ fragments and their ratios match quite well with the relative peak heights of the corresponding EICs of pure standard carbamazepine. In this way, it turns out that we can group all signals from isotopic peaks, adducts, and different charge states of a single compound at the MS^1^ level, as well as all mass fragments for every eluting compound at the MS^2^ level, using a bilinear factor decomposition method. This can be achieved without initial preprocessing steps including background correction and retention time shift alignment, considering the high selectivity of LC-MS signals and corresponding high quality of initial estimates of LC profiles using the pure variable detection approach [21, 24, 29, 40, 41]. However, due to the extensive flexibility of MCR-ALS, additional constraints can be added to the process if a higher quality of profiles is required in tricky situations, provided the data structure meets the required criteria (see below). Figure 2d also reports the MS^2^ spectrum of carbamazepine under the same instrumental conditions (CE of 30 eV) in ddMS^2^ mode. A comparison between the resolved MS^2^ spectrum in AIF mode with the current method and the ddMS^2^ profile shows a similarity score of 80% and increased relative sensitivity of the main mass fragment 194.0964 and two other fragments to the precursor ion 237.1017 when the measurement is carried out in AIF mode. The following cases further illustrate the benefits of the proposed method. An example is naproxen (MCR component 11) for which the correct information on its MS^1^ spectrum and precursor [M+H]^+^ (*m/z* 231.016) was not immediately clear due to in-source degradation. However, with this method and through global componentization, we were able to successfully recover the true MS^1^ pattern of naproxen, with the most abundant fragment ion at *m/z* 185.0960, and a straightforward (manual) assignment to its MS^2^ spectrum, which was further confirmed by mzCloud. Another example is gemfibrozil, where through a pairwise comparison with its corresponding retrieved MS^2^ profile (MCR component 14), the most intense MS^1^ peak (*m/z* 273.1459) was manually assigned to the [M+Na]^+^ adduct as the precursor ion to be further followed for annotation purposes in validation data sets. Figure 3 shows a comparison between acetaminophen and gemfibrozil with precursor ions [M+H]^+^ and [M+Na]^+^ in their retrieved MS^1^ spectra and their associated resolved MS^2^ AIF spectra.

However, as mentioned before, one significant consideration in the modeling of fused LC-MS/MS data is that LC profiles in both modes of MS data acquisition should be synchronized and span the same retention time range [29]. Although this requirement has generally been met in the current data sets, there have been some instances of distortion. For example, processing fused data matrices in the retention time range of 10 to 11 min can be explained in more detail. Here, proper recovery of MS^2^AIF for fluoxetine can be considered as a challenging case. Figure S7a–b illustrates the EICs of highly co-eluting carbamazepine and fluoxetine in their characteristic ions in MS^1^ and MS^2^ data acquisition modes. A substantial difference between the ion ratios of these chemicals due to their different ionization efficiency and fragmentation behavior is clear. The significantly lower abundance of MS/MS AIF fragments for fluoxetine and the smaller peak width relative to the MS^1^ peak, which is most likely the result of strong over-fragmentation of the AIF precursor ion [42], would lead to a discrepancy in LC windows of fluoxetine in row space of fused MS^1^ and MS^2^ data matrices and prevent efficient MS^2^ spectrum recovery. This was further confirmed by individual MCR-ALS modeling of CWA data matrices LC-MS^1^ and LC-MS^2^, following proper alignment of chromatograms and adding trilinearity constraints to the processing workflow (Fig. S7c–d). However, following row-wise concatenation and modeling of these two data blocks, while the MS^1^ spectrum of this compound was effectively recovered (due to the high purity of initial estimates and the complementary role of MS^1^ data matrix in the resolution process), its MS^2^ profile was mainly characterized by carbamazepine fragmentation patterns with a base MS/MS fragment of 194.0964 (Fig. S7e–f). This is while the recovered MS^2^ AIF spectrum of fluoxetine, through MCR-ALS modeling of LC-MS^2^ data, showed a similarity score of 96.8% with its corresponding pure standard (Fig. S7g–h). This type of issue can be tracked and checked in a real non-target data set by analyzing the LC-MS^1^ and LC-MS^2^ data in a fused and non-fused way and comparing the results.

Consequently, the simultaneous curve resolution of full-scan MS^1^ and MS^2^ AIFs in the fused method provides a two-sided advantage by directly linking the acquired MS^2^ profiles with their MS^1^ spectral counterparts (and responsible precursor ions). It can be considered for the complementary information of each resolved component and facilitates the proper assignment of MS^1^ and MS^2^ spectra. Finally, the recovered MS^2^ fragments and their ratios for the most prominent ions were compared with the EICs from the original data in Xcalibur software. The similarity scores were greater than 99% for all the target compounds. The provided MS^2^ AIF spectra along with LC and MS^1^ information obtained through modeling of global data set I (Table S1) were then considered as reference chemical information for proof of concept in validation water samples.

### Multivariate curve resolution of LC-HRMS/MS validation data set

As the first set of validation samples, the non-spiked and all spiked tap water samples with chemical standards were arranged as a global RCW augmented data matrix and each subjected to an individual CWA modeling of LC-MS^1^ and LC-MS^2^ and finally a fused LC-MS^1^-MS^2^ data modeling with MCR. Data showed chromatographic regions with different co-elution degrees with matrix components, retention time shifts, and varying drifting patterns and intensities of background signals. The number of components in the models with optimal performance was 40, 36, and 40, respectively. Therefore, fused data modeling shows a clear advantage over individual MS^2^ data modeling, since it captures more components in a non-target assay. Moreover, since the MCR model has an inherent property of swapping the positions of components in individual CWA models, each resolved component (or target) must be assigned separately. Using MCR modeling of row-wise concatenated data matrices, this issue was also resolved. The results of global modeling, including the matrix dimension, model performance parameter, resolved chromatographic profiles, and variation in recovered peak areas for 40 components across different samples, are provided in Table S2, Fig. S8, and Fig. S9, respectively. The resolved components include the target standards, background signals, and unknown matrix components. Figure S8 shows the co-elution issues (with matrix components) for some of the target compounds and also presents the resolved MS^2^ AIF spectra using the global model for acetaminophen, testosterone, and gemfibrozil. Their similarity scores with the reference spectra are 90.7%, 96.9%, and 95.4%, respectively. In fact, in addition to the significance of dealing with co-elution issues in NTA of water samples, the presence of background signals with different drifting patterns throughout the chromatographic runs can be decisive in recovering highly qualified mass spectral profiles through MCR analysis of raw data matrices. The mentioned issues are especially pertinent for the annotation of trace amounts of pollutants. However, the advantage of extended MCR-ALS similar to other multi-way methods is that it can comprehensively combine the analysis of several samples in experimental series. When different samples with varying concentrations of chemicals are simultaneously subjected to the method, the ability to detect trace peaks increases, and a more robust and reliable estimate of the pure chromatographic and spectral profiles can be obtained [43, 44]. Additionally, the current workflow can be modified to include background correction of data matrices as a preprocessing step, whenever necessary [32].

**Fig. 4:**
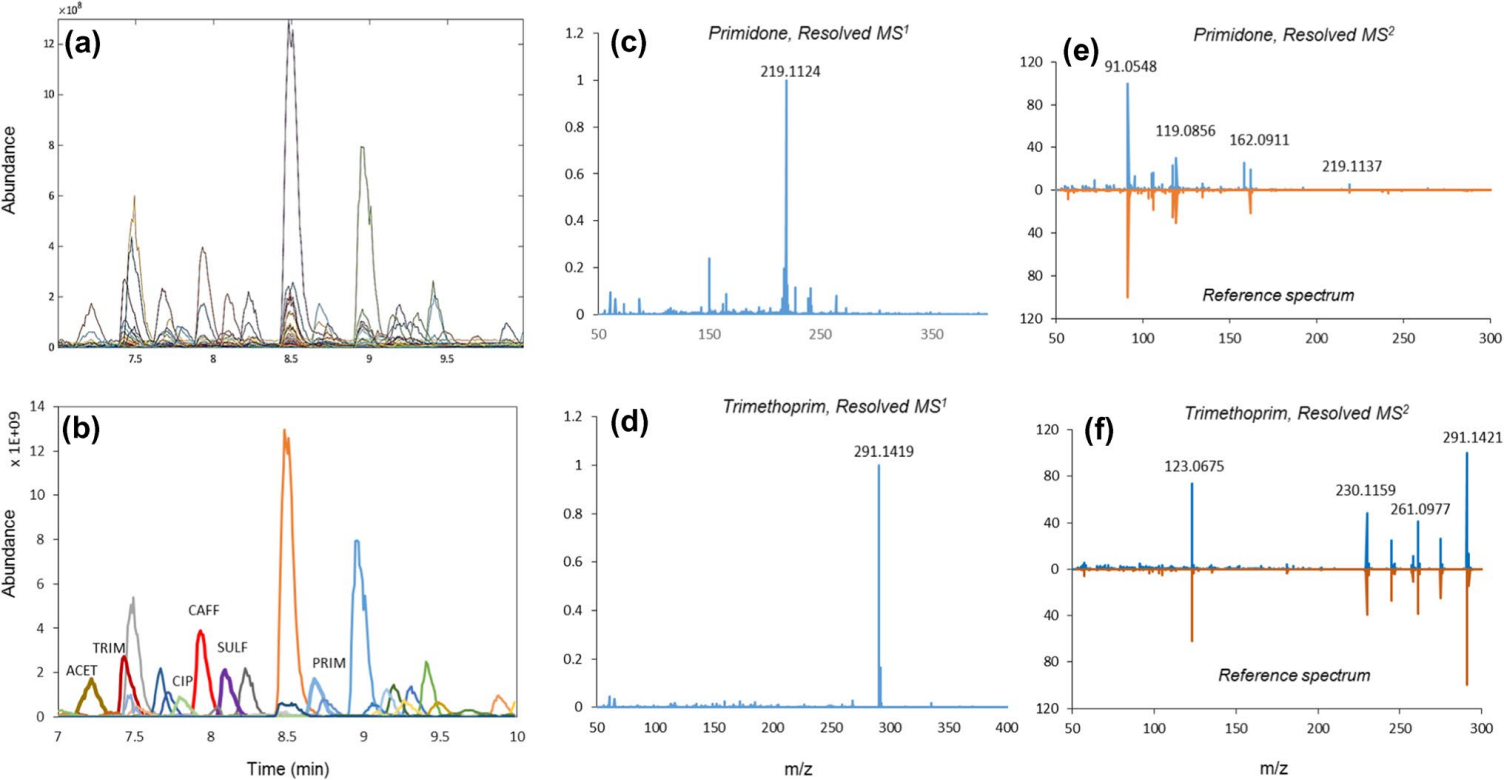
Representation of a subset of validation river water samples from data set III (**a**), corresponding resolved chromatographic profiles (**b**), resolved MS^1^ spectra of primidone (**c**) and trimethoprim (**d**) and their corresponding MS^2^ AIF spectra (**e**) and (**f**) using MCR-ALS modeling of global RCW augmented data matrices (fused model)

At the end, highly qualified MS^2^ spectra were recovered for 13 chemical standards with similarity matches ≥82% (Table S3). Thus, a global MCR-ALS model for fused data sets was effective in recovering highly qualified and interference-free MS^2^ spectra for most of the components with the current setup. The utility of the proposed methodology was further assessed on a set of extracted (and pre-concentrated) river water samples, representing a "high-level" matrix complexity, spiked with different concentration levels of target chemicals (data set III). Figure 4a shows an example of the curve resolution process for a subset of the global data matrix (7–10 min) including five target compounds. This LC interval reaches the optimal solution (*R*^2^=99.2) with 31 components under non-negativity constraint, including five target compounds and 26 background and unknown river water chemicals. The complexity of the data matrix is clear from Fig. 4b, which shows the resolved LC profiles, representing various co-eluting patterns between target chemicals of interest and other unknown matrix components. The successfully resolved MS^1^ and MS^2^ AIF spectra for primidone and trimethoprim are shown in this figure, representing match scores of 82.4% and 98.1% with reference spectra, respectively. The curve resolution process for other LC regions, including eight chemical standards, was also successful. Detected main mass fragments and their relative abundance were in accordance with Table S1 for standard samples, with match quality scores ranging from 88.2% (for acetaminophen) to 99.2% (for carbamazepine).

We encountered a challenging case in recovering the estrone MS^2^ profile due to its low abundance in the current setup experiment, where it co-eluted with highly abundant compounds in most of the samples. This complicates the correct identification, with MS^2^ match of around 60% (using the bilinear factor model), whereas other target pollutants in the extracted river water with the same concentration range had a promising MS^2^ quality score (>80%). Thus, it can be concluded that the quality of resolved MS^2^ spectra in a non-target water environment depends on contaminant classes (MS/MS fragment sensitivity) and variation patterns across samples. Nevertheless, the main advantage of MCR-ALS for chromatographic data is the flexibility to implement constraints even for a single peak in a matrix, to obtain more qualified mass spectra. As a result, different levels of model complexity can be covered when real-world situations in NTA of water samples with various degrees of complexity are encountered [31]. For example, in the mentioned case, following a careful alignment of chromatographic data and implementation of trilinearity constraint for estrone, the contribution of the main interfering compound (with the mass fragment 98.9845) to the estrone profile was effectively removed, and a more qualified MS^2^ spectrum with similarity index of 86% with the corresponding pure sample was recovered (Fig. S10). Further studies are currently underway to automatically accommodate these capabilities in modeling fused MS^1^-MS^2^ data in different scenarios.

At the end of the analysis of data set III with the global fused data sets, we tried to tentatively identify the remaining unknown MCR components through MS^1^ and MS^2^ AIF spectral profiles connected to each recovered LC profile. The final results of identification for 10 MCR components resolved in the global fused model for data set III are presented in Table S4. Figure S11 shows the results of the resolution process for MCR unknown components 9 and 23, identified as benzotriazole and 4-acetamido-antipyrine in the river water sample (data set III), respectively.

Moreover, the fused models were employed for individual modeling of row-wise fused LC-MS^1^-MS^2^ data sets (Fig. S2) using two extracted river water samples with different spiked contamination levels (100 and 10 μg/L). Basically, MCR models can be fit to each individual sample, as MCR does not require three-dimensional data. This allows the identification of elution patterns in individual modeling of samples. Our final findings regarding similarity scores for target standard compounds in modeling of single LC-MS^1^-MS^2^ measurements are presented in Table S3. It is clear that for a higher spiked level in the final extracted (pre-concentrated) sample (100 μg/L), except for fluoxetine and estrone, the similarity scores are higher than 78%. This is while in a low spiked level (10 μg/L), the quality scores of most of the chemicals are less than 20% (for individual modeling of one sample). This result supports the importance of simultaneous modeling of multiple LC-MS^1^-MS^2^ measurements after matrix augmentation [45, 46] to reduce MCR model errors and ambiguities and provide high-quality resolved MS^2^ AIF spectra. This is especially important for contaminants showing low sensitivity or low abundance or that are highly suppressed due to matrix effects in the water environment. In fact, simultaneous data processing of one or different sets of water samples is truly in line with real-world aquatic NTS advancements and perspectives. This methodology can be extremely useful in analyzing surface water samples collected at various times or locations, wastewater samples undergoing chemical or biological treatment, samples for chemical source tracking studies, and water samples measured under a variety of extraction protocols/instrumental conditions.

### Non-target screening of surface water samples

The data processing strategy for simultaneous modeling of MS^1^ and MS^2^ data sets was further utilized as the end stage of a non-target screening workflow (sample set IV), as an application example. The details of the preliminary curve resolution and multivariate data processing steps are provided in SI-7 and supplementary Figs. S12 to S15.

**Fig. 5:**
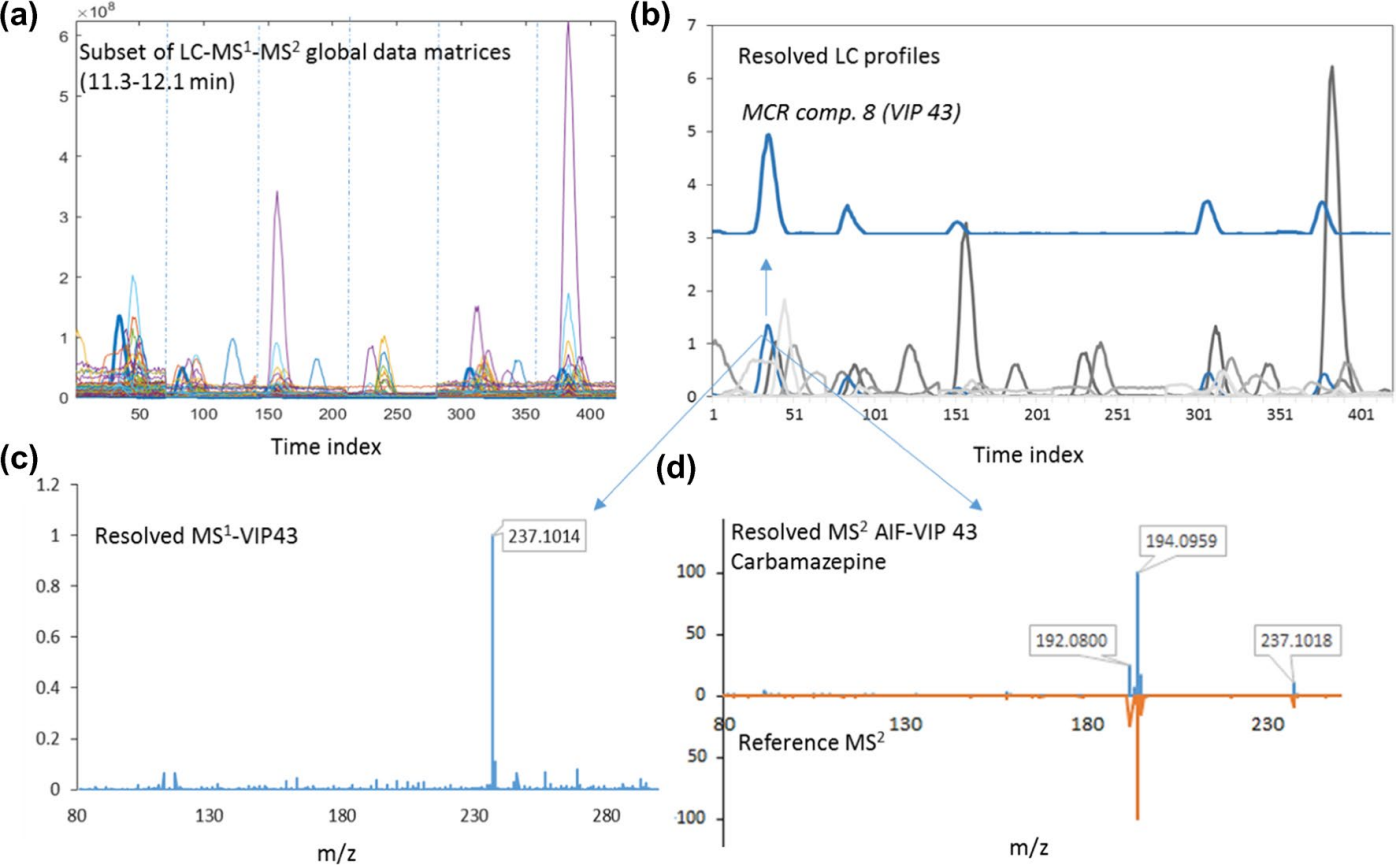
Representation of LC-MS^1^-MS^2^ global data matrices (6 water samples) for LC interval 11.3–12.1 min (**a**), corresponding resolved chromatographic profiles including carbamazepine (MCR component 8, variable importance in projection [VIP] 43) and 18 other components by MCR-ALS modeling of fused data (data set IV) (**b**), resolved MS^1^ (**c**), and MS^2^ AIF resolved and reference spectra for carbamazepine. The resolved LC profile of carbamazepine is also shown separately in subplot (**b**)

Each component associated with the second group of surface water samples was initially assigned to its corresponding resolved elution profile by MCR modeling of the original CW LC-MS^1^ data set. Then, different fused LC-MS^1^-MS^2^ data matrices (Fig. 1) were made using the LC windows including the prioritized pollutants according to their location in the chromatograms. As an overview of whole patterns, Fig. S16 shows TICs for surface water sample WS-18 (sampling site 5, Pirbazar River) in both data acquisition modes together with total ion mass currents for MS^1^ and MS^2^-AIF modes. For instance, for prioritized component 43, an LC window of 11.3–12.1 min was extracted throughout the whole data set of LC-MS^1^ and LC-MS^2^ and subjected to global modeling by MCR-ALS. Figure 5 shows the results of this processing for a subset of raw data from sampling sites 3 to 5 for carbamazepine-positive assignment. Among other prioritized pollutants, we were able to annotate six compounds using the mzCloud database, and the rest of the compounds could not be identified (Table S6). The identified chemicals could be attributed to various classes, such as caffeine, carbamazepine (and its primary metabolite carbamazepine-10,11-epoxide), dextromethorphan, piperine, and buphedrine (the urinary metabolite of buphedrone, a drug of abuse), which are mainly released to the environment throughout non- or insufficiently treated domestic, hospital or industrial wastewater effluents [47, 48]. Also, the presence of the herbicide bensulfuron-methyl could be attributed to the agricultural runoffs. Its main application purpose is to control broadleaf weeds in rice paddies [9, 49], and rice is the most important agricultural product of Gilan province. Overall, the identified chemicals could be considered anthropogenic contaminants that were released into the river water via wastewater or non-point runoffs.

## Conclusion

In the current study, global MCR modeling of fused full-scan MS^1^ and MS/MS (AIF) data sets using LC-HRMS/MS measurements has been proposed as a highly efficient approach for enhancing the performance of DIA-based workflows for non-targeted analysis of trace contaminants in surface water samples. With the integration of MS^2^ AIF data matrices to initial concatenated LC-MS^1^, precursor ions in the resolved MS^1^ spectrum can be directly linked to their corresponding resolved MS/MS spectra, both associated with their unified LC profiles. This facilitates the detection and identification of prioritized contaminants, especially when simultaneous analysis of multiple chromatographic data is considered. Moreover, the implementation of the extended MCR-ALS strategy for simultaneous MS-based data analysis is flexible, expandable, and customizable according to study needs and data structure. Further, the use of a unified model reduces data analysis time and improves results accuracy by modeling fused data sets.

We believe that while this methodology addresses some key needs for highly effective annotation of non-targeted LC-MS/MS AIF data, it still has room to accommodate combined strategies for a more comprehensive capture of chemical space in non-targeted pollution screening studies.

### Supplementary information

ESM 1 (DOCX 2052 kb)

## Abbreviations

DIA-AIF: Data-independent acquisition–all-ion fragmentation
LC-HRMS: Liquid chromatography coupled to high-resolution mass spectrometry
EICs: Extracted ion chromatograms
PCA: Principal component analysis
SVD: Singular value decomposition
MCR-ALS: Multivariate curve resolution–alternating least squares
OPLS-DA: Orthogonal partial least squares–discriminant analysis
VIP: Variable importance in projection
NTA: Non-target analysis
