# Supplementary material for: Enhanced efficiency of MS/MS all-ion fragmentation for non-targeted analysis of trace contaminants in surface water using multivariate curve resolution and data fusion
Source: Anal Bioanal Chem. 2024 Jan 11;416(5):1165–77. doi: 10.1007/s00216-023-05102-x (PMC10850027; doi:10.1007/s00216-023-05102-x)
Supplement: Supplementary file 1 — (DOCX 2052 kb) [file 216_2023_5102_MOESM1_ESM.docx]

Supplementary Material

**Enhanced efficiency of MS/MS all ion fragmentation for non-targeted analysis of trace contaminants in surface waters using multivariate curve resolution and data fusion**

Maryam Vosough^1,2*^, Amir Salemi^1^, Sarah Rockel^1^ , Torsten C. Schmidt^1,3^

*^1^ Instrumental Analytical Chemistry and Centre for Water and Environmental Research (ZWU), University of Duisburg-Essen*, *Universitatsstr. 5, Essen 45141, Germany*

*^2^Department of Clean Technologies, Chemistry and Chemical Engineering Research Center of Iran, Tehran, Iran, P.O. Box 14335-186 Tehran, Iran*

*^3^IWW Water Centre, Moritzstr. 26, Mülheim an der Ruhr 45476, Germany*

^*^Corresponding author. [maryam.vosough@uni-due.de](mailto:maryam.vosough@uni-due.de)

**Table of contents**

S1: Target chemical compounds

S2: Details of experimental methods (Set III)

S3: Experimental method and data processing (samples set IV)

S4: MCR-ALS resolution of LC−HRMS/MS water data

S5: Data matrices for global data modeling and validation

S6: Modeling of LC-MS^1^, LC-MS^2^ and LC-MS^1^-MS^2^ (sets I to III)

S7: Results of nontarget screening of surface waters (set IV)

**S1: Target chemical compounds**

**Table S1.** Overview of standard chemicals with MS^1^ and MS^2^ m/z values, retention time and molecular formula^1^.

| **Compound name** | **Molecular Formula** | **Theoretical (m/z) MS^1^ Precursor** | **Retention**  **time (min)** | **Relative mass error (ppm)** | **MS^2^ Top m/z Peaks^2^** | **MCR profile** |
| --- | --- | --- | --- | --- | --- | --- |
| Acetaminophen | C8H9NO2 | 152.0706 | 6.75 | 0 | 152.0705  111.0442  110.0602  92.0500  65.0394 | 5 |
| Caffeine | C8H10N4O2 | 195.0877 | 7.44 | 0.51 | 195.0877 138.0658  110.0699 83.0610 | 2 |
| Carbamazepine | C15H12N2O | 237.1022 | 10.14 | 3.79 | 237.1017  195.0997  194.0960  192.0807 | 4 |
| Ciprofloxacin | C17H18FN3O3 | 332.1405 | 7.26 | 0.30 | 333.1433  332.1404  288.1503  245.1037 | 20 |
| Trimethoprim | C14H18N4O3 | 291.1451 | 6.93 | 0.68 | 291.1447  275.1134  261.098  245.1037  230.1160  123.0666 | 3 |
| Gemfibrozil | C15H22O3 | 273.1467  [M+Na]+ | 15.59 | 2.96 | 273.1459  129.0911  123.0807  83.0862  73.0656  55.0552 | 14 |
| Fluoxetine | C17H18F3NO | 310.1413 | 10.11 | 0.64 | 310.1411  289.1796  259.0841  238.1055  148.1120  60.0816 | 24 ^3^ |
| Sulfamethoxazole | C10H11N3O3S | 254.0594 | 7.58 | 1.57 | 156.0111  108.0446  99.0558  92.0500 | 9 |
| Ibuprofen | C13H18O2 | 207.138 | 15.72 | 1.93 | 161.1324  119.0858  105.0703  56.9656 | 15 |
| Naproxen | C14H14O3 | 231.1016 | 13.39 | 1.73 | 185.0960  170.0725  153.0698  154.0772 | 11 |
| Primidone | C12H14N2O2 | 219.1128 | 8.16 | 0 | 162.0913  119.0858  117.0702  106.0655  91.0548 | 7 |
| Progestrone | C21H30O2 | 315.2319 | 14.68 | 0.31 | 315.2318  298.2242  109.0651  97.0652 | 6 |
| Testosterone | [C_19_H_28_O_2_](https://pubchem.ncbi.nlm.nih.gov/#query=C19H28O2) | 289.2162 | 12.88 | 0.69 | 311.1979  289.2159  109.0651  97.0652 | 8 |
| Estrone | C18H22O2 | 271.1693 | 12.33 | 0.37 | 253.1585  197.0961  159.0805  133.0648  105.0036 | 21 |

^1^MS^1^ precursors and MS^2^ fragments have been obtained using MCR-ALS resolution of D_global-fused_ data for standard solution of pure standard chemicals.

^2^ MS^2^ fragments and their ratios have been confirmed with the extracted ion chromatograms (EICs) from the original data in Xcalibur software.

^3^ Fluoxetine cannot be identified using MCR-ALS resolution of RCWA data. Only, MS^1^ m/z value of the precursor ion is recovered. The MS^2^ fragments were obtained through modeling of CWA of LC-MS^2^ data matrices and manually confirmed with the EICs in Xcalibur software.

**S2: Details of experimental methods (Set III)**

**S2-1: Sample Preparation**

For sample preparation step, the water samples (1000 mL) were extracted on Oasis HLB (Waters, Germany) cartridge (preconditioned: 2 × 5 mL methanol and 2 × 5 mL water, LC-MS grade). The cartridges were rinsed (2×10 mL of HPLC water), dried under vacuum (15 min), and eluted (5×5 mL methanol, LC-MS grade), and the solvent was evaporated to complete dryness at 50 °C under a gentle nitrogen stream. Then, the samples were re-dissolved in 10 mL HPLC waters and all validation samples were provided by spiking the proper aliquots of mixed standard solutions into the extracted samples.

**S2-2: LC-HRMS/MS measurements**

The non-target screening was conducted by using a Dionex UltiMate 3000 HPLC system (Thermo Scientific, Bremen, Germany). An Orbitrap mass spectrometer (QExactive Thermo Scientific, Bremen, Germany) with electrospray ionization in positive mode was used for mass spectrometric measurements.

The mobile phase consisted of 0.1% formic acid (A) and methanol with 0.1 % formic acid (B) and the chromatographic column was XSelect HSS T3 (2.1 mm x 75 mm, 3.5 μm particle size), which was supplied from Sigma Aldrich. The flow rate was set at 0.3 mL min^-1^. The initial Eluent composition is 100 % A and 0 % B, which was held like that for 2 minutes. From two to 4 minutes, the composition was changed to 50 % A and 50 % B. From 4 minutes to 17 minutes, it was changed to 2 % A and 98 % B. This composition was held for 5 minutes, until 22 minutes. From 22 minutes to 22.1 minutes, the composition was changed back to the initial composition of 100 % A and 0 % B, which was held until 30 minutes (the end of the run). An Orbitrap mass spectrometer (QExactive Thermo Scientific, Bremen, Germany) with electrospray ionization in positive mode was used for mass spectrometric measurements in the following conditions: spray voltage, 4000 V, capillary temperature 350 °C, sheath gas flow rate 40, aux gas flow rate 5 (au), sweep gas flow rate 10 (au), S-Lens RF level 60, and aux gas heater temperature 100 °C. Samples were measured in Full Scan MS^1^ and MS^2^ AIF in positive (ESI) mode in a range 50-750 m/z with resolution of 70,000 FWHM at 200 m/z and ACG Target 1e6 and 3e6, and Maximum IT 100 and 1000 ms, respectively. Higher energy collisional dissociation (HCD) was obtained using a normalized collision energy (NCE) of 30 eV.

**S3: Experimental method and data processing (samples set IV)**

**S3-1: Sampling**

The samples had been collected from five different river basin in north of Iran. 4 L of surface water sample was collected in each point. Two upstream samples were collected from the Sefidrud River basin, the second-largest river in Iran. Sample 1 was grabbed at the Sefidrud Dam (N 36° 45' 9.939", E 49° 23' 18.384"). Sample 2 belonged to Shahrud River (N 36° 37' 8.5722", E 49° 32' 29.2806"), which merges with Sifidrud, at the Sefidrud Dam. Both samples could represent the quality of the water, just before the impact of the highly populated rural and urban areas starts. Also, limited industrial discharges and agricultural runoffs are expected prior to the sampling points. Sample 3 was collected from downstream of the Sefidrud River (N 37° 25' 32.6496", E 49° 54' 46.5762"), where the river then passes a National Park (Bojagh) and connects the Caspian Sea. The river up to this point receives a considerable amount of untreated sewage and also agricultural runoff. Sample 4 belongs to the Zarjub River (N 37° 19' 24.8874", E 49° 33' 36.2232"), which (along with another river, the Goharrud River) passes through Rasht, the largest city in the North of Iran. Despite the presence of the wastewater treatment plant and sewage collection network, the river still receives a large amount of wastewater, both from the city of Rasht and the rural areas in its vicinity. Sample 5 was grabbed from the Pirbazar River (N 37° 20' 36.7872", E 49° 32' 49.2606"). This river is formed by merging two previously mentioned rivers (Zarjub and Goharrud) and also receives the wastewater treatment plant effluent of Rasht (through Goharrud) and ends in the Anzali Lagoon.

**S3-2: Extraction procedure**

Water samples were collected in pre-cleaned 4 L amber glass bottles, stored at 4°C, and extracted within 2 days. The replicate samples were filtered using 1.2 μm and 0.7 μm glass fiber filters (Whatman^TM^) and extraction of the organic compounds was conducted within 48 h after sampling using SPE with Strata-X polymer cartridges (Phenomenex, USA) containing 500 mg of polymer adsorbent in 6 mL tubes. Briefly, the cartridges were preconditioned by 5 mL methanol and 5 mL acetone, equilibrated (2 × 5 mL water, LC-MS grade), were loaded with 600 mL sample (containing 0.1% W/V Na_2_EDTA) at a flow rate of approximately 2.5 mL min^-1^, were rinsed with 6 mL Milli-Q water, dried under vacuum and finally stored at -18 °C until further sample preparation. The second round of sample extraction was conducted on the water samples by adjusting the pH of the samples to 2 and replacing the water (containing EDTA) throughout the cartridge conditioning and rinsing with HPLC water with pH=2. In all cases, the elution and solvent evaporation to dryness was performed using 6 mL acetone and 6 mL methanol (LC-MS grade) and a gentle stream of nitrogen stream at 50 °C, respectively. Samples were shipped frozen to Germany on dry ice and arrived frozen. Before analysis, the samples and laboratory blanks were re-dissolved in 1 mL mobile phase. All samples were measured in one batch with pooled quality control samples (containing 100 ng of surrogate standards) injected every three samples. Further, method blanks were processed the same way as described above with ultra-pure water instead of samples.

**S3-3: LC-HRMS/MS measurements**

LC-HRMS/MS measurements for nontarget screening was performed using the same LC-Q-Orbitrap system as mentioned in section 2.2 of main text. The mobile phase consisted of eluent A: ultrapure water + 0.1% formic acid, and eluent B: methanol + 0.1% formic acid (both MS grade). Injection volume was 20 µL. The chromatographic column was Atlantis T3 (2.1 mm x 150 mm, 3 μm particle size). The flow rate was set at 0.3 mL min^-1^. The eluent condition for set IV was similar to the rest of the samples. Samples were measured in Full Scan MS^1^ and MS^2^ AIF in positive (+) mode in a range 80-1000 m/z with resolution of 70,000 and ACG Target 3e6 and 1e6, and Maximum IT 100 and 1000 ms, respectively. MS/MS activation type of HCD was applied using a normalized collision energy (NCE) of 30 eV. Mass calibration was performed with the calibration solution (Pierce LTQ Velos Positive/Negative Ions Calibration Solution, Thermo Scientific, Bremen, Germany).

**S3-4: Data processing**

Individual ROI matrices were constructed for each subset of LC/MS measurement in MS^1^ full scan mode. To this end, following data compression for each ‘peak array’, each produced data matrix was segmented in three chromatographic segments and then the simultaneous compression of all data sub-matrices were conducted. This was initiated by compression of data sub-matrices to find their common and uncommon ROI m/z and continued in a similar way with the rest of sub-matrices. At the end, three global data matrix, (**D_Aug_CW_MS_1**) containing the blank samples were built in a column-wise augmented way for each chromatographic segment and submitted to extended MCR-ALS modeling, according to Fig. S1 (a). The output MCR-ALS modeling on each **D_Aug_cw_** is three matrices **C_Aug_cw_,** **S^T^** and peak areas, which are the pure LC profiles for the resolved components, corresponding resolved MS^1^ profiles and the quantification indexes, respectively. The peak area values of the final matrices were then transferred to a peak area Table whose columns and rows corresponded to the water samples and resolved MCR-ALS components, respectively. According to the criteria outlined in previous studies, the irrelevant and undesired MCR-ALS components together with common detected components in method blanks were removed before statistically analyzing peak areas and MCR component patterns [1, 2]. Then, before multivariate statistical modeling and in order to account for heteroscedasticity in the data and systematic changes in average intensity levels across different experimental conditions, a logarithmic transformation was conducted on the data A_aug_, followed by normalization (row-wise) and autoscaling. The final cleaned peak area Table was then subjected to PCA and OPLS-DA for data exploration and prioritization step.

**S4: MCR-ALS resolution of LC−HRMS/MS water data**

In the current investigation, MCR-ALS was utilized to decompose all sets of arranged validation samples and complex chromatographic data from water samples into their corresponding pure LC profiles and MS^1^ and MS^2^ mass spectral profiles, without inputting any priori information. Fig. S1 illustrates the non-fused decomposition process for column-wise augmented matrices for each LC-MS^1^ and LC-MS^2^ AIF. For simultaneous decomposition of LC-MS^1^ and LC-MS^2^ data, augmentations in the m/z dimension for each sample can also be considered, as shown in Fig. S2. Fig. 1 illustrates the final globalization of data augmentation in sample mode and MS^2^ data acquisition mode.


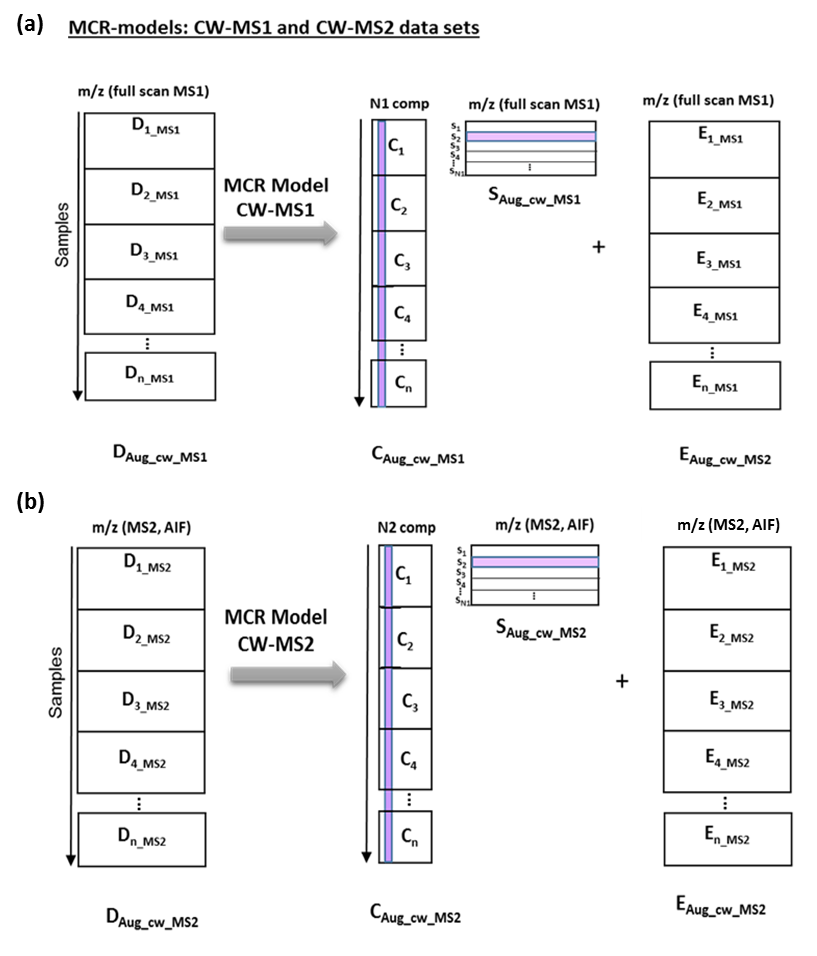


**Fig. S1**. Representation of individual modeling of column-wise augmented (CW) LC-MS1(a) and LC-MS2 (b) data arrangement and MCR resolution of each set of samples.

**
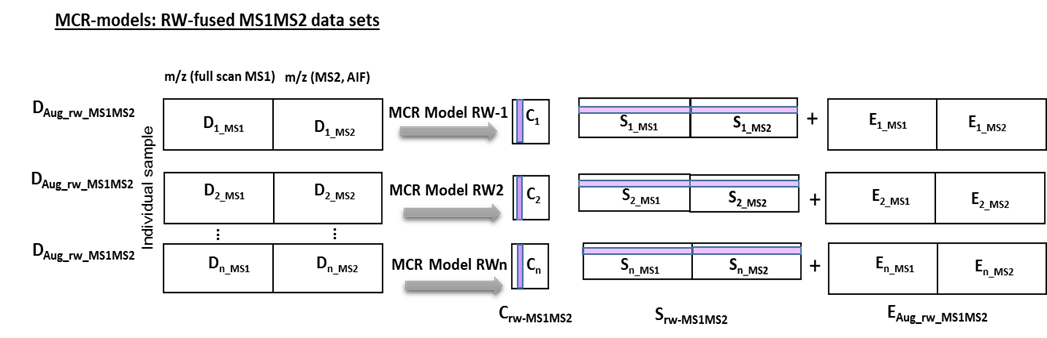
**

**Fig. S2**. Representation of row-wise (RW) fused LC-MS^1^-MS^2^ data arrangement and MCR resolution for each individual sample.

**S5: Data matrices for global data modeling and validation**

**Table S2.** LC-HRMS/MS matrices and MCR-ALS quality results for global modeling of fused MS^1^ and MS^2^ data sets.

| Sample type-data arrangement | Time region | Retention time (min) | NC^1^ | Size of **D_glob-fused_**  (I×K, J) | R^2 (2)^ | Lof^3^ |
| --- | --- | --- | --- | --- | --- | --- |
| - Set I: Mixed standards in pure water samples. | whole | 5.30-18.27 | 25 | 9006×1920 (MS^1^:1070,MS^2^:850) | 99.4 | 6.5 |
| - Set II: Non-spiked and spiked tap water (TW^4^) sample and direct injection | whole | 5.30-18.27 | 40 | 9006×2464 (MS^1^:1474,MS^2^:990) | 99.6 | 7.2 |
| - Set III: Non-spiked and spiked river water (RW^5^) sample and SPE^6^-based procedure | I | 7.02-9.98 | 31 | 2352×5676  (MS^1^:3506,MS^2^:2170) | 99.2 | 8.8 |
|  | II | 10.00-14.98 | 25 | 3920×5676 | 98.8 | 10.7 |
|  | III | 13.58-16.69 | 25 | 2450×5676 | 99.2 | 9.0 |
| - Set III: Spiked river water sample (RW7) 100 µg/L (SPE-based procedure) | I | 7.02-9.98 | 31 | 336×5676 | 99.6 | 5.8 |
|  | II | 10.00-14.98 | 25 | 560×5676 | 99.5 | 6.9 |
|  | III | 13.58-16.69 | 25 | 350×5676 | 99.5 | 6.3 |

^1^ Number of components

^2^ Explained variance (%), R^2^=100 × $\frac{\sum_{i=1}^{m} \sum_{j=1}^{n} d_{i,j}^{2}-\sum_{i=1}^{m} \sum_{j=1}^{n} e_{i,j}^{2}}{\sum_{i=1}^{m} \sum_{j=1}^{n} d_{i,j}^{2}},$ where d_ij_ and e_ij_ represent each element of the measured data and residual data matrices, respectively.

^3^ Lack of fit (%), Lof (%) = 100 × $\frac{\sum_{i=1}^{m} \sum_{j=1}^{n} e_{i,j}^{2}}{\sum_{i=1}^{m} \sum_{j=1}^{n} d_{i,j}^{2}}$

^4^ Tap water

^5^ River water

^6^ Solid phase extraction

**S6: Modeling of LC-MS^1^, LC-MS^2^ and LC-MS^1^-MS^2^ (sets I to III)**


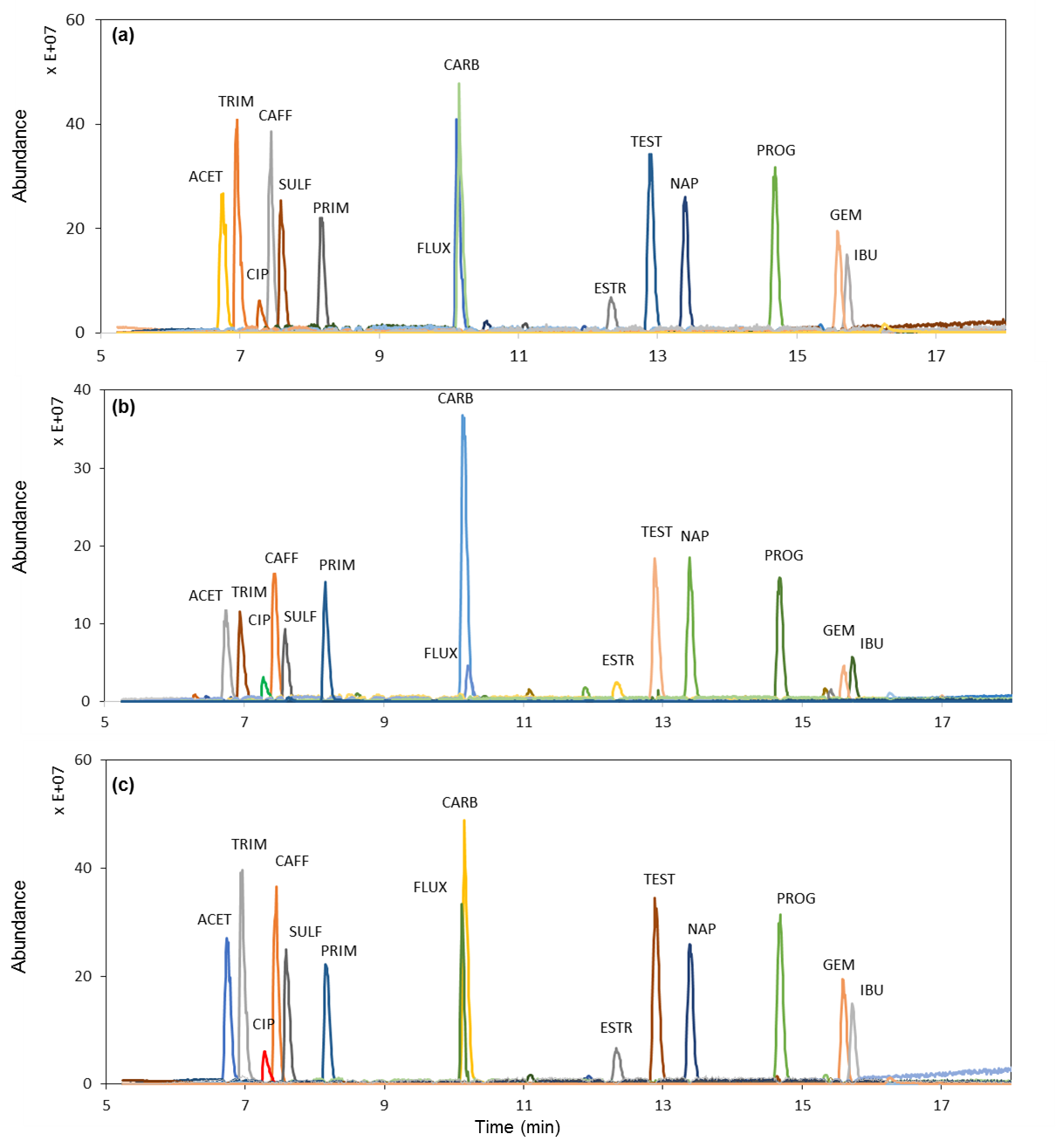


**Fig. S3.** Retrieved LC profiles for a subset (100 µg/L) of the mixed standard solutions (set I samples) by extended MCR-ALS modeling of individual CWA LC-MS^1^ data (a), CWA LC-MS^2^ data (b), and RCWA LC-MS^1^-MS^2^ data sets (c).


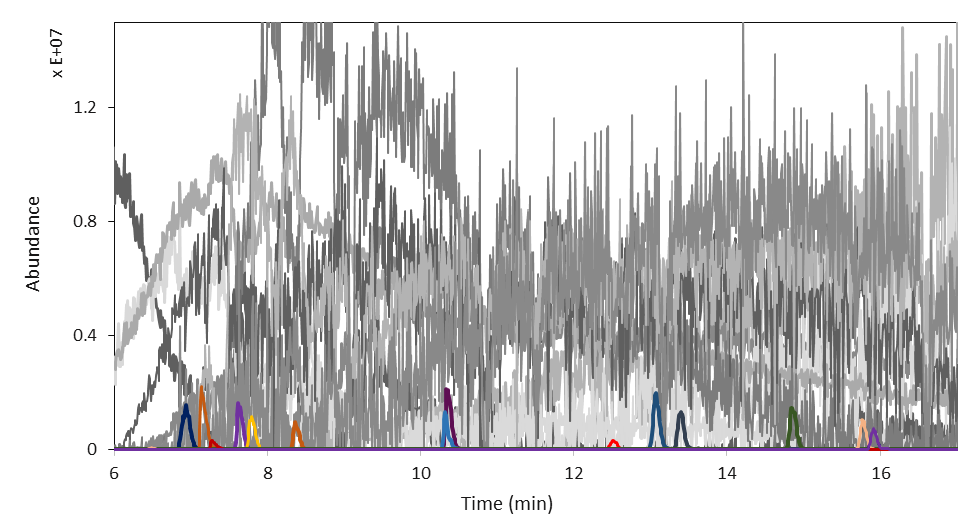


**Fig. S4.** Visualization of resolved LC profiles for target compounds and background components (grey lines) for the mixed standard solution (0.5 µg/L) through extended MCR-ALS modeling of RCWA LC-MS^1^-MS^2^ data set I. Target compounds can be followed according to retention times in Table S1 and Fig. S3.


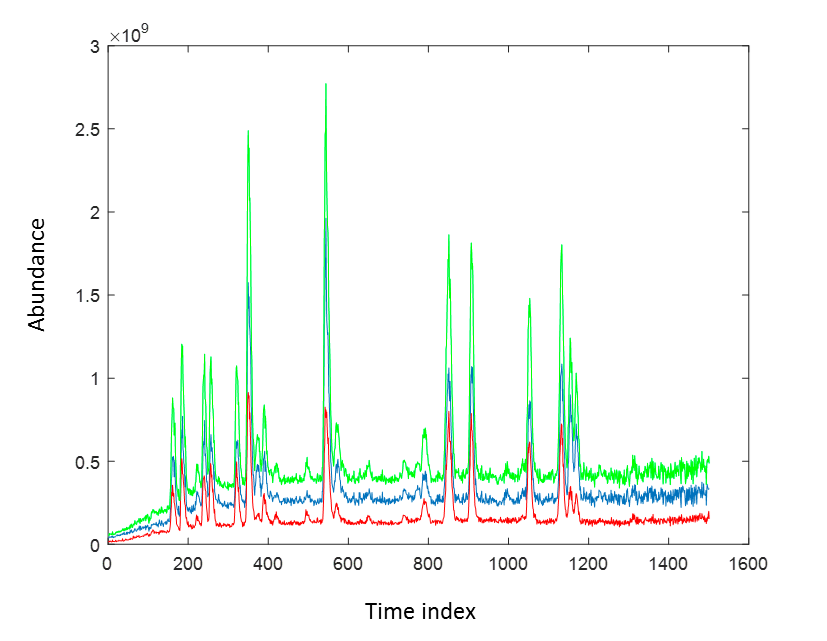


**Fig. S5.** Visualization of total ion chromatograms (TICs) in MS^1^ (blue line), MS^2^-AIF (red) and MS^1^-MS^2^ modes (green line) for the target compounds (10 µg/L) spiked in water sample.

**
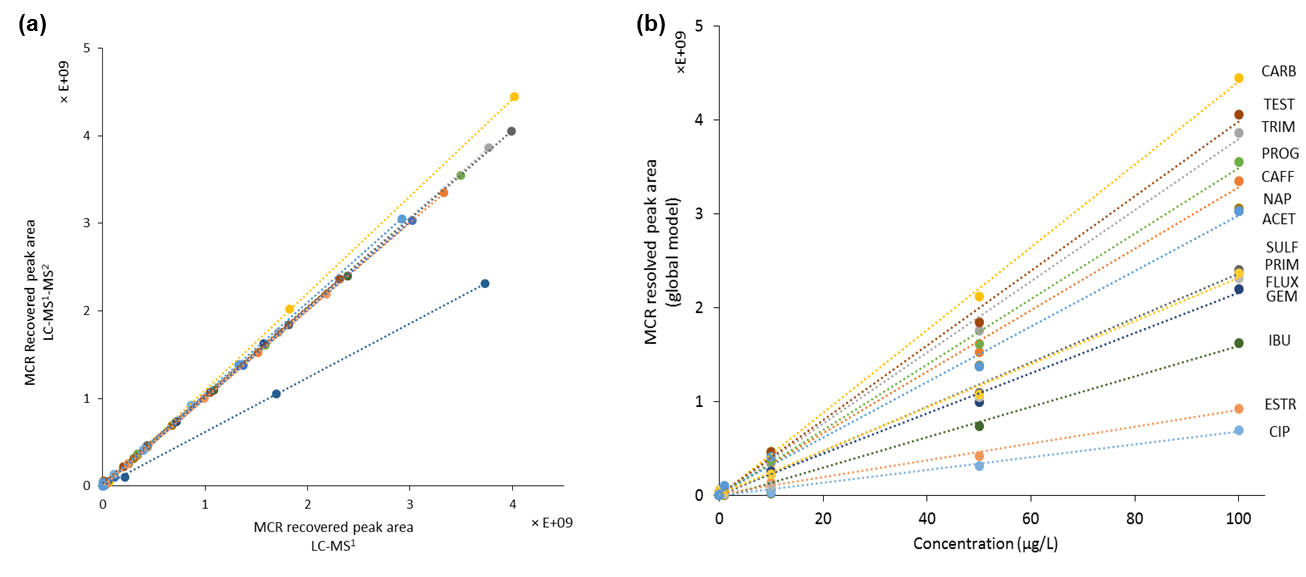
**

**Fig. S6.** Representation of (a) variation of MCR-ALS recovered peak areas for the target chemicals using RCWA LC-MS^1^-MS^2^ data against CWA LC-MS^1^, (b) regression plots for the resolved peak areas of the pure target chemicals using RCWA LC-MS^1^-MS^2^ data for concentration range 0.1-100 µg/L.

**
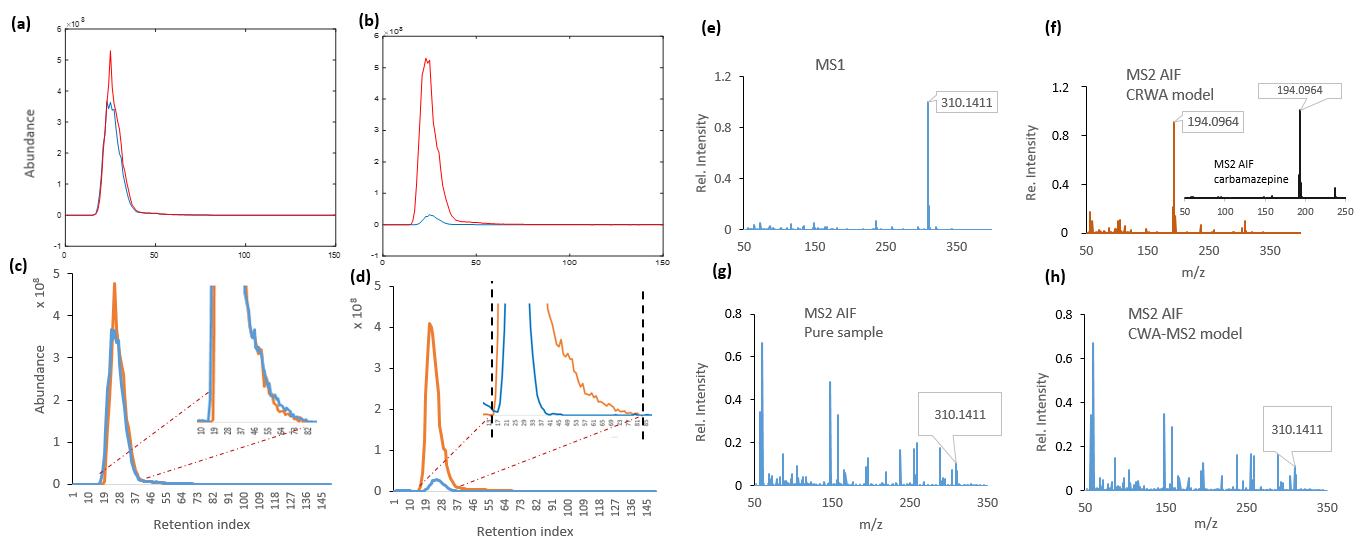
**

**Fig. S7.** Representation of EICs in MS^1^ (red lines) and MS^2^ (blue lines) of highly coeluting (a) carbamazepine (at m/z 237.1016 and m/z 194.0964) and (b) fluoxetine (at m/z 310.1411 and m/z 148.1120). Retrieved LC profiles of carbamazepine (c) and fluoxetine (d) by individual MCR-ALS modeling of CWA LC-MS^1^ (red lines) and LC-MS^2^ (blue lines) data matrices, respectively. Recovered MS^1^ (e) and MS^2^ AIF (f) spectra for fluoxetine through processing of fused LC-MS^1^-MS^2^ data for data set (I). MS^2^ AIF spectra of standard sample of fluoxetine obtained through MCR-ALS modeling of CWA LC-MS^2^data matrices for a set of (g) pure standard samples and (h) data set (I), respectively. The insert plot in (f) illustrates MS^2^ AIF spectrum of standard sample of carbamazepine.

**
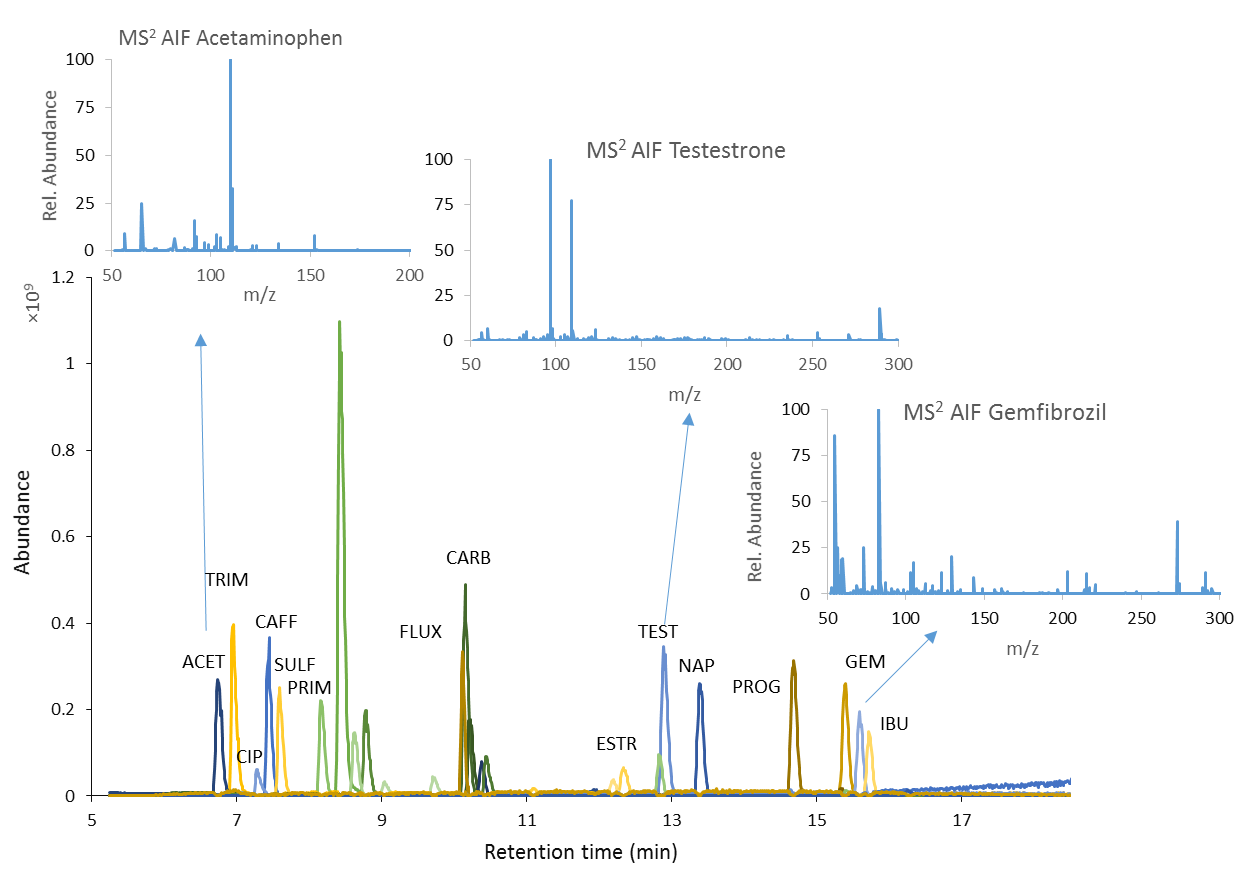
**

**Fig. S8.** Retrieved LC and MS^2^ AIF profiles for spiked tap water sample (100 µg/L) through fused LC-MS^1^-MS^2^ data set II by extended MCR-ALS modeling.

**
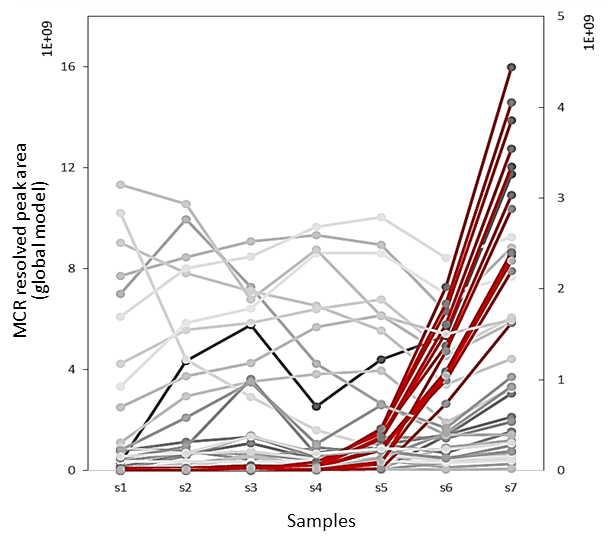
**

**Fig. S9.** Representation of variation of recovered peak areas of 40 resolved components (using global MCR model) in tap water analysis, including target compounds (red lines), background signals and unknown sample constituents (grey lines) across different samples (spiked-in concentration range 0.1-100 µg/L).

**Table S3.** Similarity scores obtained for target compounds using MCR modeling of fused LC-MS^1^-MS^2^ validation data sets.

| Compound name | Tap water  Global model | River water  Global model | River water  Single sample (spike level 100 µg/L)^1^ | River water  Single sample (spike level 10 µg/L)^1^ |
| --- | --- | --- | --- | --- |
| Acetaminophen | 90.7 | 88.2 | 88.7 | <20 |
| Caffeine | 99.7 | 93.5 | 95.9 | 78.6 |
| Carbamazepine | 99.5 | 99.2 | 98.9 | 76.4 |
| Ciprofloxacin | 98.0 | 93.2 | 97.8 | <20 |
| Trimethoprim | 98.7 | 98.1 | 94.2 | <20 |
| Gemfibrozil | 95.4 | 92.9 | 94.2 | <20 |
| Fluoxetine | NA^2^ | NA | NA | NA |
| Sulfamethoxazole | 90.5 | 89.8 | 90.1 | <20 |
| Ibuprofen | 93.2 | 92.8 | 78.3 | <20 |
| Naproxen | 98.3 | 97.4 | 97.9 | <20 |
| Primidone | 86.4 | 82.4 | 80.6 | 27.5 |
| Progestrone | 96.8 | 99.1 | 98.6 | <20 |
| Testosterone | 96.9 | 98.8 | 99.0 | 68.6 |
| Estrone | 82.0 | 61.2 | 55.7 | <20 |

^1^ Spiked concentrations in final extracted (SPE pre-concentrated) river water sample.

^2^ Not applicable, since the prerequisite before row-wise augmentation of MS^1^ and MS^2^ data matrices was not met.


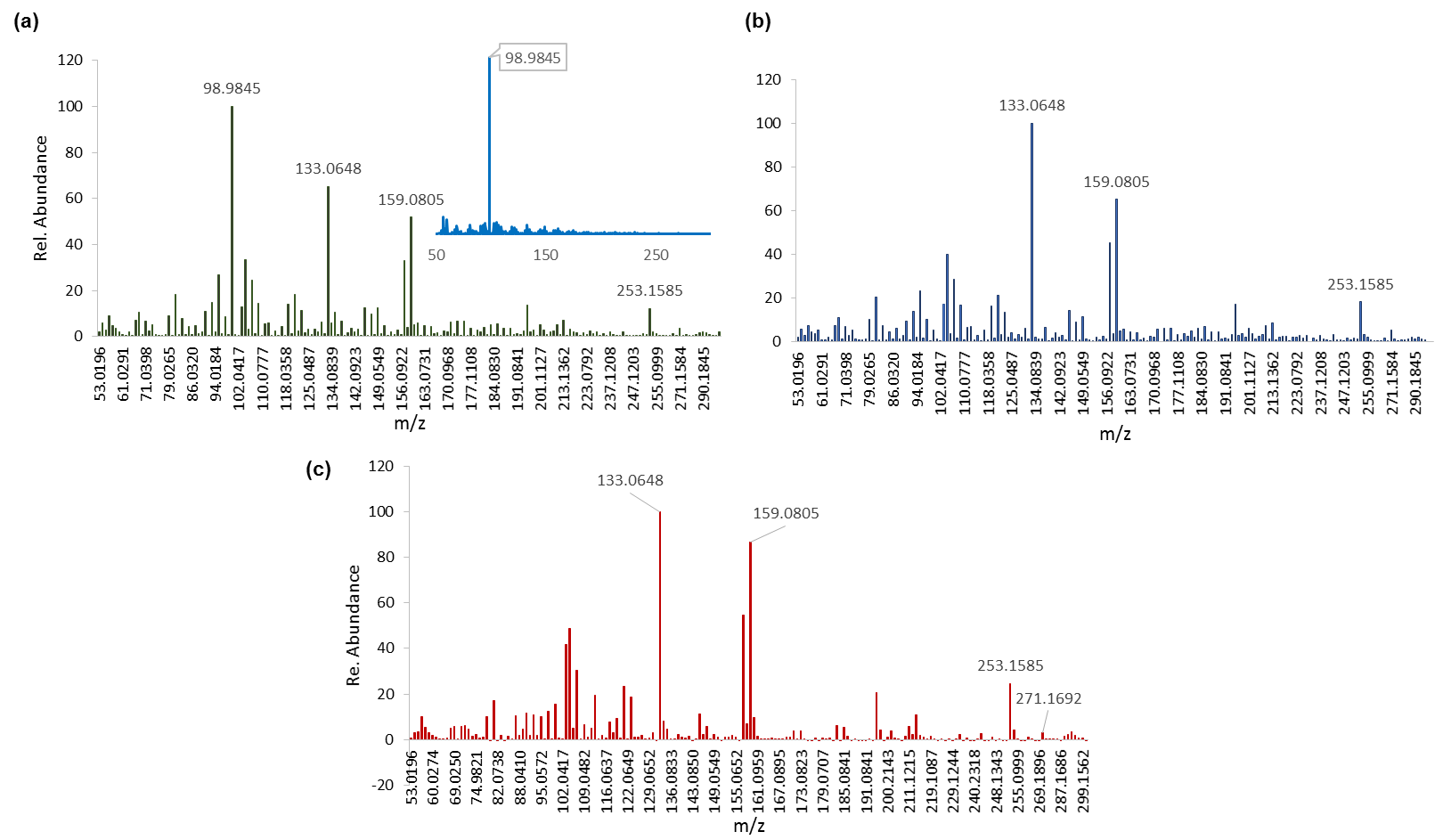


**Fig. S10.** Representation of resolved MS^2^ AIF spectra for estrone through MCR-ALS modeling of fused LC-MS^1^-MS^2^ data set III (second subset) for bilinear decomposition model (a), trilinear decomposition model for estrone (b), and MS^2^ AIF spectrum of standard sample of estrone (c). The insert plot in (a) illustrates MS^2^ AIF spectrum of interfering compound.

**Table S4.** Identification results for MCR components resolved in global fused model for data set III.

| MCR component  (Global model) | Exact mass | Retention time (min) | Compound name | Theoretical (m/z) | Molecular Formula | Relative mass error (ppm) | MS^2^ Top m/z Peaks | Identification level |
| --- | --- | --- | --- | --- | --- | --- | --- | --- |
| C7_r1 | 232.1077 | 7.6 | 4-Antipyrine-Formylamino | 232.10805 | C12H13N3O2 | 1.2 | 214.0972  159.0915  146.0598  104.0497  83.0609  56.0503 | tentative |
| C9_r1 | 120.0556 | 8.2 | Benzotriazole | 120.05562 | C6H5N3 | 0.2 | 120.0547  92.0499  65.0393 | confirmed |
| C26_r1 | 181.0716 | 7.5 | Paraxanthine | 181.07200 | C7H7N4O2 | 2.2 | 181.0722  152.0452  142.0616  124.0497 | tentative |
| C23_r1 | 246.1229 | 7.7 | 4-Acetamido-antipyrine | 246.12370 | C13H15N3O2 | 3.2 | 228.1129  204.1127  159.0916  104.0497  83.0609  56.0503 | tentative |
| C12_r1 | 134.0711 | 9.2 | 4/5-methyl-1H-benzotriazole | 134.07127 | C7H7N3 | 1.3 | 134.0697  105.0450  95.0495  79.0548 | tentative |
| C22_r1 | 165.1021 | 9.9 | N’1-phenylpropanohydrazide | 164.09496  (monoisotopic mass)  165.1021 | C9H12N2O | - | 109.1014  108.0684  92.0499  65.0393 | tentative |
| C2_r2 | 274.2734 | 12.4 | Palmitic Acid | 256.24023  (monoisotopic mass)  274.2742 | C16H32O2 | - | 274.2735  256.2631 | tentative |
| C18_r2 | 223.0949 | 11.6 | Diethyl phthalate | 223.09649 | C12H13O4 | 6.8 | 149.0231  121.0285  65.03933 | tentative |
| C17_r3  C1_r3 (window subset) | 301.1408  279.1583 | 15.9 | Dibutyl phthalate | 301.1410  [M+Na]^+^  279.1591  [M+H]^+^ | C16H22O2 | 0.6  2.8 | 150.0265  149.0231  121.0285  65.0393  57.0707 | tentative |
| C15_r3 | 198.1277 | 14.3 | Dibenzylamine | 198.12773 | C14H14N | 0.3 | 91.0548  65.0393 | tentative |

**
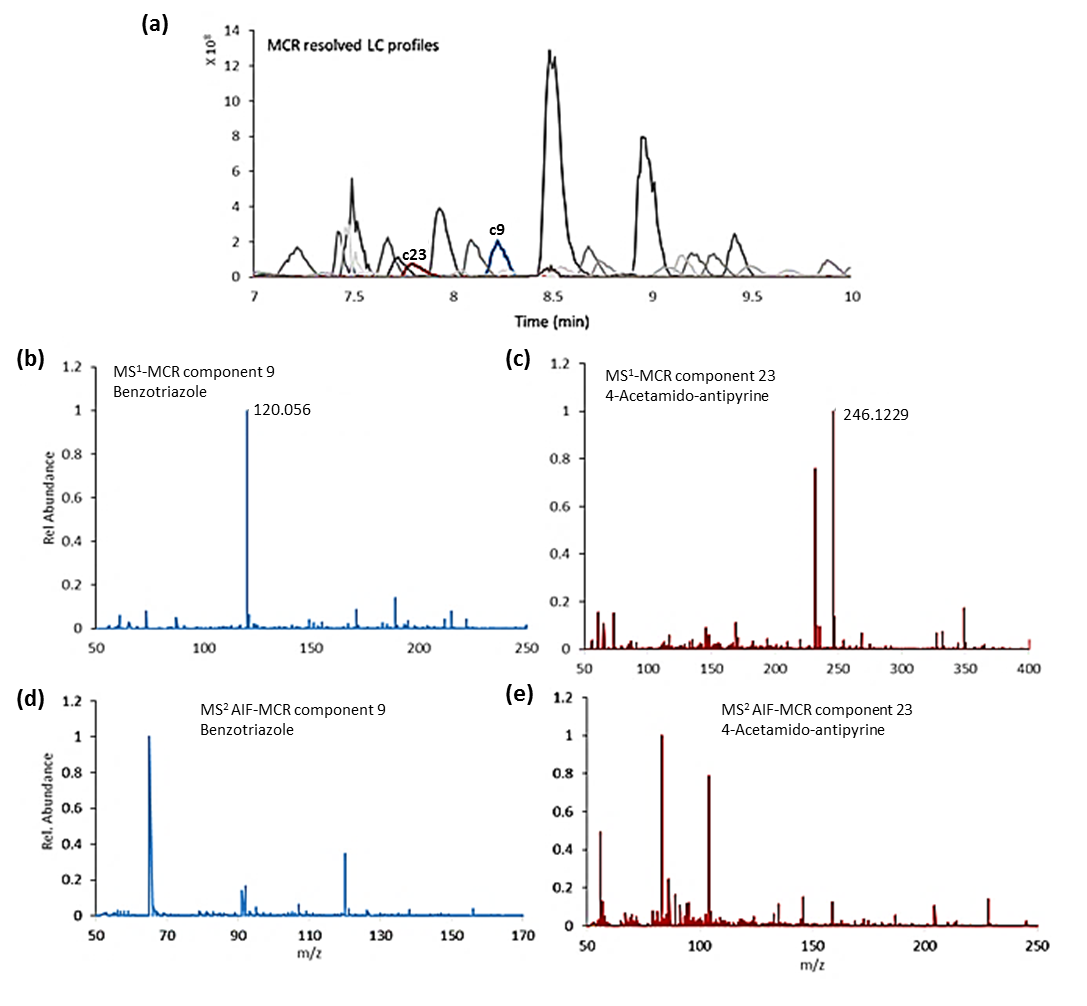
**

**Fig. S11.** Representation of resolution process for MCR unknown components 9 and 23, identified as benzotriazole and 4-acetamido-antipyrine in the river water sample (data set III), respectively, (a) shows the retrieved LC profiles in retention time window 7-10 min, (b) and (c) present the resolved MS^1^ profiles, (d) and (e) present MS^2^ AIF profiles for components 9 and 23, respectively.

**S7: Results of nontarget screening of surface waters (set IV)**

**S7-1: Initial data processing**

Hierarchically, this data set includes two groups of upstream (8 samples) and downstream (12 samples) sites, each group representing two different SPE conditions with two lab replications. Fig. S12 shows total ion chromatograms (TICs) in full scan MS^1^ for ten extracted surface waters (set IV), following initial MCR processing of LC-MS^1^ data matrices (see SI-5-4). Chromatographic regions I to III were resolved using 64, 70, and 82 components, respectively. The percentage of explained variance (R^2^) for all models were at least 95% and the lack of fit (LOF) values were 6%, which are acceptable considering the complexity of the data sets. Therefore, a total number of 216 MCR-ALS components were used to explain the variance of the whole data set. This number includes all detected species, solvent contributions, noisy signals, artifacts, and background signals. Then, a matrix cleaning was carried out and all nonrelevant components with no reliable chromatographic shape and spectral features were removed according to the criteria in ref [2]. The final results indicated that 92 out of the 216 resolved MCR components were related to organic pollutants while the rest were considered less formative and unknown background signals. The final cleaned data matrix (20×92) was subjected to multivariate methods for further exploration and prioritization step.

**
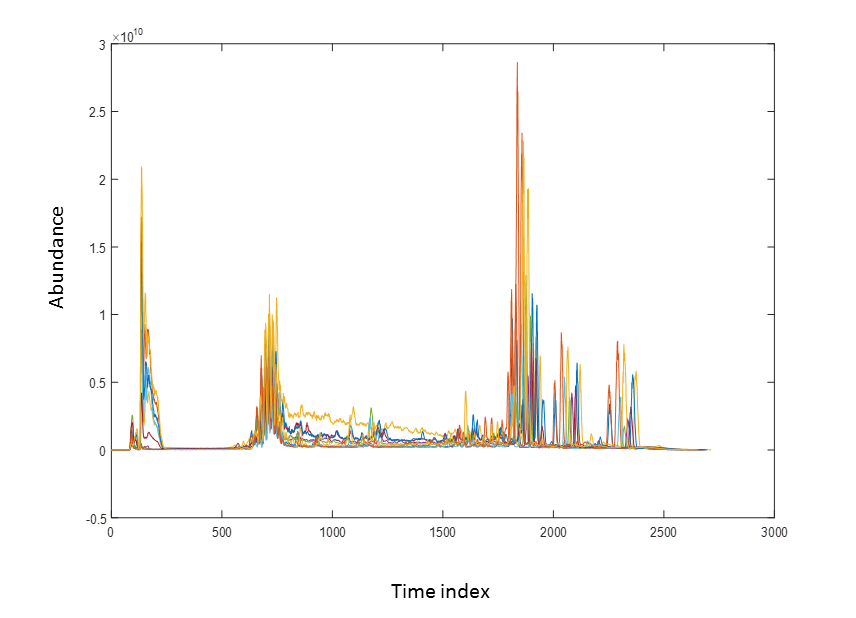
**

**Fig. S12.** TICs in full scan MS^1^ for ten surface waters (set IV).

**S7-2: PCA and OPLS-DA model results based on LC-MS^1^ resolution by MCR**

As an initial data exploration, PCA was applied on the matrix of peak area values to investigate the inherent structure of the data. The score plot for the first two PCs presented in Fig. S13 shows a separation of two major groups of surface water samples. Group 1 consisted data matrices (WS1-8) provided from two upstream samples of the Sefidrud River (sampling sites 1 and 2) and group 2 consisted all data matrices (WS9-20) provided from downstream of the Sefidrud River, Zarjub River and Pirbazar River (sampling sites 3 to 5, respectively). Moreover, peaks could be tentatively characterized on the basis of water groups using their loading values. A further subgrouping of the less polluted samples associated with two sampling sites (red markers) is evident, which is hardly observed for the more polluted samples due to a higher within-class dispersion pattern caused by extraction conditions.

As a result of the considerable variation in sampling locations in two main groups, OPLS-DA, a well-known supervised learning method, was applied to the peak areas data matrix in order to identify the components that showed a significant difference among the chosen sites. By using this method, all peak areas (predictor variable) are correlated with a vector containing the sample type class membership (predicted variable). Moreover, a quantitative estimation of the discriminatory power of each MCR component by means of VIP (variable importance in projection) values can be obtained [3]. Fig. S14 shows the more relevant variables in the VIP score plot for two classes of water samples using an  OPLS-DA model with LV1 (31.6%) and LV2 (43.5%) on data with R^2^ of 97.3% and Q2 of 91.2% and class error (CV) zero. VIPs with values greater than one define which of the obtained components was more influential in distinguishing the upstream samples of the Sefidrud and the rest of the highly polluted surface water samples. In this case, out of the total number of 92, 44 components were found with VIPs>1, of which 27 pollutants are found to be highly relevant with samples of group 2 compared to group 1. Fig. S15 shows the occurrence patterns (as recovered MCR peak areas) of six prioritized pollutants obtained by OPLS-VIP modeling of LC-MS^1^ data set IV. Components 43 and 54 positively assigned to carbamazepine and piperine using fused LC-MS^1^-MS^2^ MCR modelling with confidence levels 1 and 3, respectively.


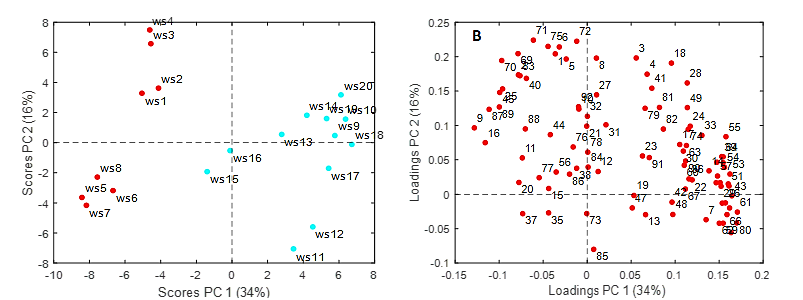


**(B)**

**(A)**

**Fig. S13**. (A) PC1 vs. PC2 scores plot obtained for sampling sites 1 and 2 (WS1-WS8, red circles) and sampling sites 3 to 5 (WS9-WS20, blue circles) surface water samples (Set IV), measured in positive ionization mode (B) Corresponding loading plot.


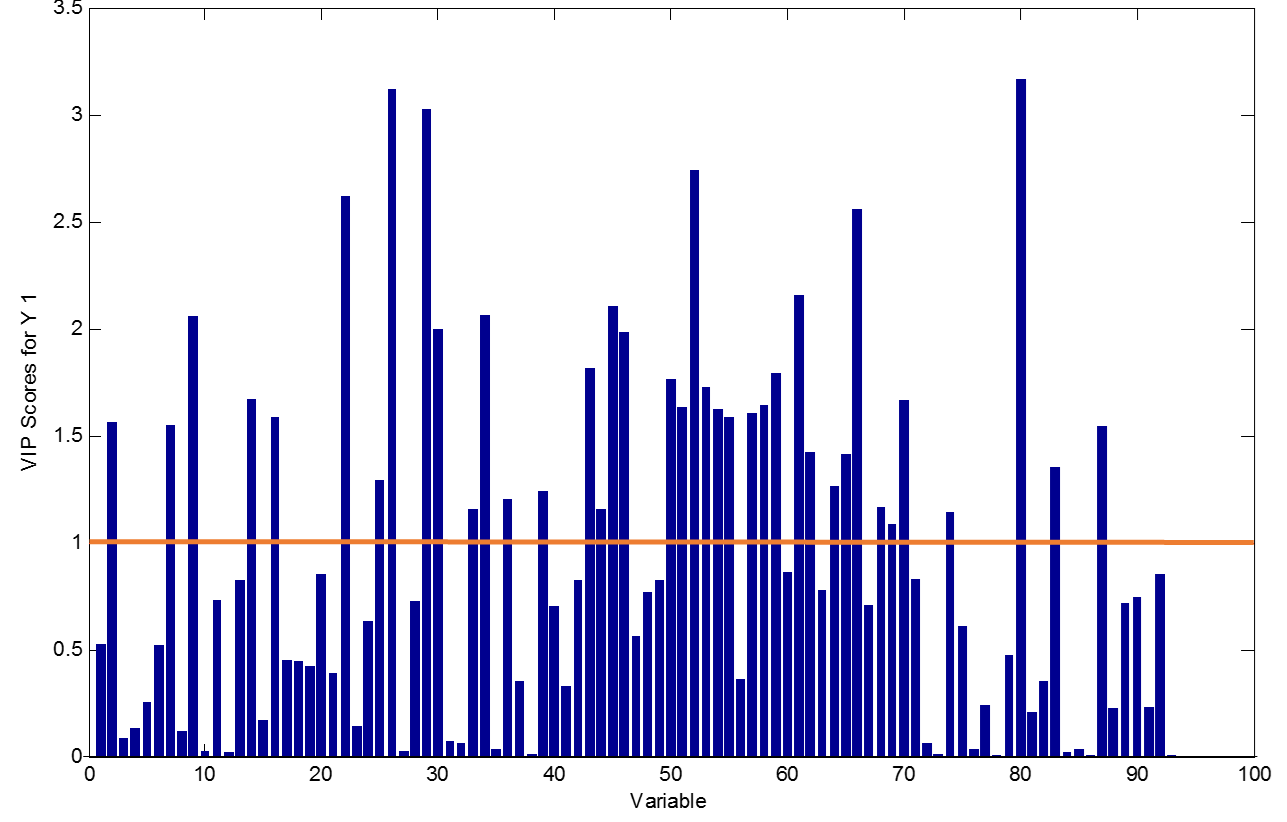


**Fig. S14.** Representation of VIP scores for each variable using the OPLS-DA model for data set IV. Horizontal red line shows the threshold value selecting the most important variables. The model was constructed with LV1 (31.6%) and LV2 (43.5%) and by performing cross validation of preprocessed data with R^2^ of 97.3% and Q^2^ of 91.2% and Class error (CV) zero.


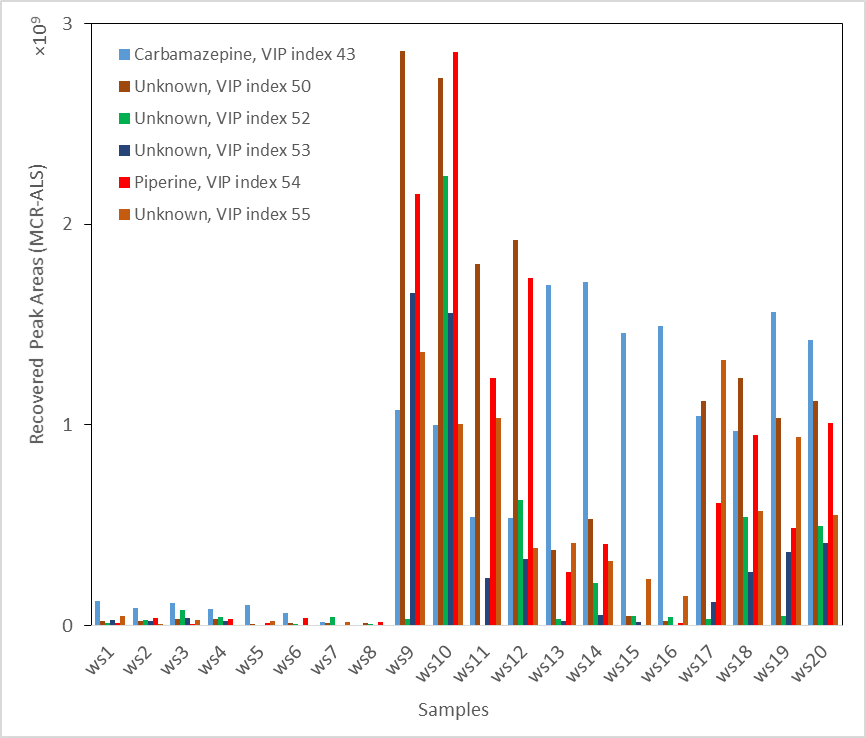


**Fig. S15.** Representation of peak area variations of six prioritized pollutants obtained by OPLS-VIP modeling of LC-MS^1^ data set IV. Components 43 and 54 positively assigned to carbamazepine and piperine using fused LC-MS^1^-MS^2^ MCR modelling with confidence levels 1 and 3, respectively.

**S7-3: Global resolution of LC-MS^1^-MS^2^ data and annotation of prioritized compounds**


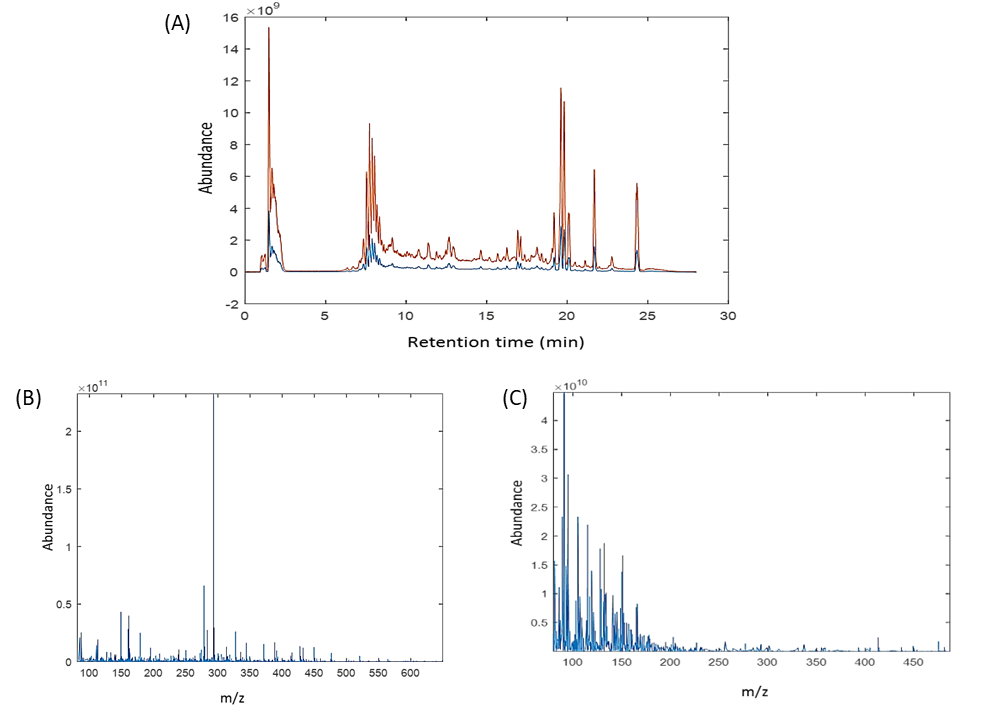


**Fig. S16.** TICs in MS^1^(red line) and MS^2^-AIF modes (blue line) (A), total ion mass currents for MS^1^ (B) and MS^2^-AIF (C) modes for surface water WS-18.

**Table S5.** Identification results for MCR components resolved in global fused model for data set IV.

| MCR component  (Global model) | Exact mass | Retention time (min) | Compound name | Theoretical (m/z) | Molecular Formula | Relative mass error (ppm) | MS^2^ Top m/z Peaks | Identification level |
| --- | --- | --- | --- | --- | --- | --- | --- | --- |
| C23_VIP14 | 272.1995 | 9.2 | Dextrometh-orphan | 272.20089 | C18H25NO | 4.8 | 272.1998  215.1423  213.1266  171.0799  159.0799  147.0799 | tentative |
| C5_VIP7 | 195.0867 | 8.2 | Caffeine | 195.0876 | C8H9N4O2 | 4.6 | 195.0867  138.0658  110.0710 | confirmed |
| C55_VIP43 | 237.1014 | 11.6 | Carbamazepine | 237.1022 | C15H11N2O | 3.4 | 237.1016  194.0959  192.0801 | confirmed |
| C82_VIP54 | 286.1425 | 15.2 | Piperine | 286.14377 | C17H18NO3 | 4.2 | 286.1441  250.1019  235.0979  201.0556  173.0955  135.0436 | tentative |
| C56_VIP46 | - | 15.9 | Buphedrine | 180.13829 | C11H17NO | - | 162.1272  147.1038  133.1007  132.0804  131.0851  117.0568  107.0852  105.0695  91.05408 | tentative |
| C58_VIP59 | 253.0957 | 9.6 | Carbamazepine 10,11-epoxide | 253.09715 | C15H12N2O2 | 5.4 | 182.0959  180.0802  169.0642  152.0616  128.0616 | tentative |
| C80_VIP66 | 411.0957 | 13.7 | Bensulfuron methyl | 411.0969 | C`6H18N4O7S | 2.9 | 182.0554  166.0858  149.0593  119.0852  91.0541 | tentative |

**References:**

1. Hohrenk LL, Vosough M, Schmidt TC (2019) Implementation of Chemometric Tools To Improve Data Mining and Prioritization in LC-HRMS for Nontarget Screening of Organic Micropollutants in Complex Water Matrixes. Anal Chem 91:9213–9220. https://doi.org/10.1021/acs.analchem.9b01984

2. Verkh Y, Rozman M, Petrovic M (2018) Extraction and cleansing of data for a non-targeted analysis of high-resolution mass spectrometry data of wastewater. MethodsX 5:395–402. https://doi.org/10.1016/j.mex.2018.04.008

3. Chong IG, Jun CH (2005) Performance of some variable selection methods when multicollinearity is present. Chemom Intell Lab Syst 78:103–112. https://doi.org/10.1016/j.chemolab.2004.12.011
